# Facile *in situ* reductive synthesis of both nitrogen deficient and protonated g-C_3_N_4_ nanosheets for the synergistic enhancement of visible-light H_2_ evolution†

**DOI:** 10.1039/c9sc05060d

**Published:** 2020-02-03

**Authors:** Weisong Li, Zheng Guo, Litong Jiang, Lei Zhong, Guoning Li, Jiajun Zhang, Kai Fan, Sergio Gonzalez-Cortes, Kuijuan Jin, Chunjian Xu, Tiancun Xiao, Peter P. Edwards

**Affiliations:** School of Chemical Engineering & Technology, State Key Laboratory of Chemical Engineering, Tianjin University Tianjin 300350 China cjxu@tju.edu.cn; Inorganic Chemistry Laboratory, University of Oxford South Parks Road Oxford OX1 3QR UK xiao.tiancun@chem.ox.ac.uk peter.edwards@chem.ox.ac.uk; Institute of Physics, Chinese Academy of Sciences Beijing 100190 China

## Abstract

A new strategy is reported here to synthesize both nitrogen deficient and protonated graphitic carbon nitride (g-C_3_N_4_) nanosheets by the conjoint use of NH_4_Cl as a dynamic gas template together with hypophosphorous acid (H_3_PO_2_) as a doping agent. The NH_4_Cl treatment allows for the scalable production of protonated g-C_3_N_4_ nanosheets. With the corresponding co-addition of H_3_PO_2_, nitrogen vacancies, accompanied by both additional protons and interstitially-doped phosphorus, are introduced into the g-C_3_N_4_ framework, and the electronic bandgap of g-C_3_N_4_ nanosheets as well as their optical properties and hydrogen-production performance can be precisely tuned by careful adjustment of the H_3_PO_2_ treatment. This conjoint approach thereby results in improved visible-light absorption, enhanced charge-carrier separation and a high H_2_ evolution rate of 881.7 μmol h^−1^ achieved over the H_3_PO_2_ doped g-C_3_N_4_ nanosheets with a corresponding apparent quantum yield (AQY) of 40.4% (at 420 nm). We illustrate that the synergistic H_3_PO_2_ doping modifies the layered g-C_3_N_4_ materials by introducing nitrogen vacancies as well as protonating them, leading to significant photocatalytic H_2_ evolution enhancements, while the g-C_3_N_4_ materials doped with phosphoric acid (H_3_PO_4_) are simply protonated further, revealing the varied doping effects of phosphorus having different (but accessible) valence states.

## Abstract 2

Nitrogen deficient and protonated g-C_3_N_4_ was fabricated by the conjoint protocol utilizing NH_4_Cl as gas template and H_3_PO_2_ as doping agent, leading to enhanced visible-light harvesting and charge carrier separation to achieve efficient H_2_ evolution.

## Graphical abstract

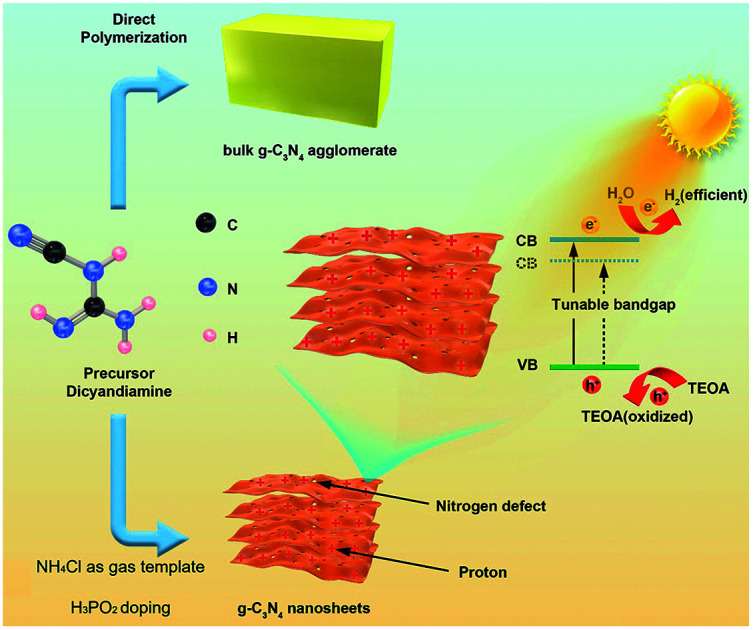

## Introduction

Graphitic carbon nitride (g-C_3_N_4_), a prototypical metal-free semiconductor, has been intensively studied owing to its excellent photocatalytic applications in H_2_ production, environment remediation, CO_2_ reduction and photosynthesis.^1–5^ Bulk g-C_3_N_4_ with high thermal, chemical and photocatalytic stabilities can be readily synthesized *via* direct thermal polymerization of nitrogen-rich precursors. However, the visible-light photocatalytic efficiency of such directly polymerized g-C_3_N_4_ material is far from satisfactory stemming from its limited visible-light absorption range, low surface area, confined active sites, and rapid charge-carrier recombination. Thus, various techniques including morphology engineering,^6,7^ constructing Z-scheme heterostructure or unique surface bonding states,^8–10^ doping with both nonmetal and metal elements,^11,12^ introducing elemental defects or vacancies and protonation have been applied in order to improve the photocatalytic performance of g-C_3_N_4_.^13–15^ Inspired by the two-dimensional graphene-like nanosheet of the material, efforts have also been made to transform bulk g-C_3_N_4_ into porous structures composing thin nanosheets which help deliver superior photocatalytic activities.^7^

Recently, it has been demonstrated that the photocatalytic activity of g-C_3_N_4_ under visible-light irradiation can be significantly enhanced by introducing nitrogen defects into the basic g-C_3_N_4_ “melon” structure as well as doping with compounds of phosphorus.^2,12^ To date, nitrogen deficient g-C_3_N_4_ was synthesized through alkali-assisted polymerization, hydrogen reduction, high-temperature thermal polymerization and hydrothermal routes.^2,13,16,17^ Unlike other methods-which routinely suffer from control difficulties-the so-called alkali-assisted polymerization achieves a highly effective control of both the type and the abundance of the introduced nitrogen defects leading to enhanced photocatalytic H_2_ evolution.^2^ However, this alkali-assisted approach, using KOH, NaOH or Ba(OH)_2_ is unlikely to work in the “bottom-up” strategy which utilizes NH_4_Cl as a so-called dynamic gas template for scalable production of g-C_3_N_4_ nanosheets, due to the non-coexistence of NH_4_Cl and alkali metal hydroxides.^7^ Moreover, it has been shown that the alkali-assisted process progressively decreases the layer stacking distance and reduces the specific surface area of g-C_3_N_4_ nanosheets when excessive KOH is added.^2^ This suggests the over-addition of alkali metal hydroxides would cause a severe decrease in photocatalytic activity because of the unfavorable crystal morphology changes.

Phosphorus, has emerged as a likely contender to be introduced into the g-C_3_N_4_ matrix to achieve enhanced photocatalytic performance. In most of the prior work,^15,18–20^ pentavalent phosphorus such as H_3_PO_4_, (NH_4_)_3_PO_4_, BmimPF_6_ ionic liquid, Na_2_HPO_4_, and CO(NH_2_)_2_·H_3_PO_4_ figures strongly as the phosphorus doping agents. To the best of our knowledge, there is little work reported on the synthesis of phosphorus-doped g-C_3_N_4_ using phosphorus compounds having lower valence states and the photocatalytic ability of such low-valent phosphorus doped g-C_3_N_4_ has hardly been studied.^4^

Here, we present a one-step, *in situ* reduction method utilizing the strategy of using NH_4_Cl as dynamic gas template and H_3_PO_2_ doping for the synthesis of nitrogen deficient g-C_3_N_4_ nanosheets. The photocatalytic activity of g-C_3_N_4_ was greatly enhanced with the formation of both protonated and porous nanosheets by using NH_4_Cl as dynamic gas template. With the co-addition of H_3_PO_4_ as dopant, g-C_3_N_4_ nanosheets were further protonated and delivered superior photocatalytic activities. However, besides protonation, the g-C_3_N_4_ nanosheets doped by the lower-valent phosphorus compound H_3_PO_2_ also led to a reduced electronic bandgap in the material and enhanced visible light absorption. The differences observed between the H_3_PO_2_ and H_3_PO_4_ doped g-C_3_N_4_ nanosheets promotes a discussion of using dopants with phosphorus at different valence states. It demonstrates the possibility of using low-valent phosphorus compounds to both synthesize and modify g-C_3_N_4_ in one single step, thereby offering a comprehensive enhanced and efficient visible-light photocatalytic performance. Thus, the preparation strategy reported here represents an effective approach to optimize the morphology, chemical composition, optical response and resulting activity of g-C_3_N_4_ photocatalysts.

## Experimental

The standard bulk g-C_3_N_4_ reference (B-CN) was prepared by the direct pyrolysis polymerization of dicyandiamide. Preliminary, 4 g dicyandiamide powder was placed in a 50 mL alumina crucible with cover and then was calcined in static air at 550 °C for 4.5 hours with a ramping rate of 2.3 °C min^−1^. The g-C_3_N_4_ nanosheets were prepared by the modified “bottom-up” strategy with NH_4_Cl as dynamic gas template,^7^ and the as prepared g-C_3_N_4_ nanosheets without and with addition of phosphorus dopants were labeled as G-CN and *M*-P*n*-CN, respectively. Here, *M* represents the phosphorus dopant usage (weight percentage of the added phosphorus element to the dicyandiamide precursor, ranging from 0.2 to 3.2) and P*n* (*n* = 1 or 2) meant that the corresponding dopant was H_3_PO_4_ and H_3_PO_2_, respectively. Firstly, 4 g dicyandiamide and 20 g NH_4_Cl were premixed with 5 mL solution containing the designed amount of dopant (for G-CN, the addition amount of dopants was zero). For example, in the preparation of 0.8-P*n*-CN samples, the addition amounts of 85 wt% H_3_PO_4_ and 50 wt% H_3_PO_2_ were 119.0 and 136.3 mg, respectively. Then, the powder and solution mixture were stirred to form homogeneous slurry. Finally, the slurry was calcined with the same procedures in B-CN preparation. All the as prepared samples were collected and stored at room temperature in normal atmosphere.

Full experimental details including reagents, characterization, transient photocurrent measurement, RTK-Solar visible-light H_2_ evolution system (Fig. S1†) and AQY measurement can be found in the ESI.†

## Results and discussion

### Morphology

**Fig. 1:**
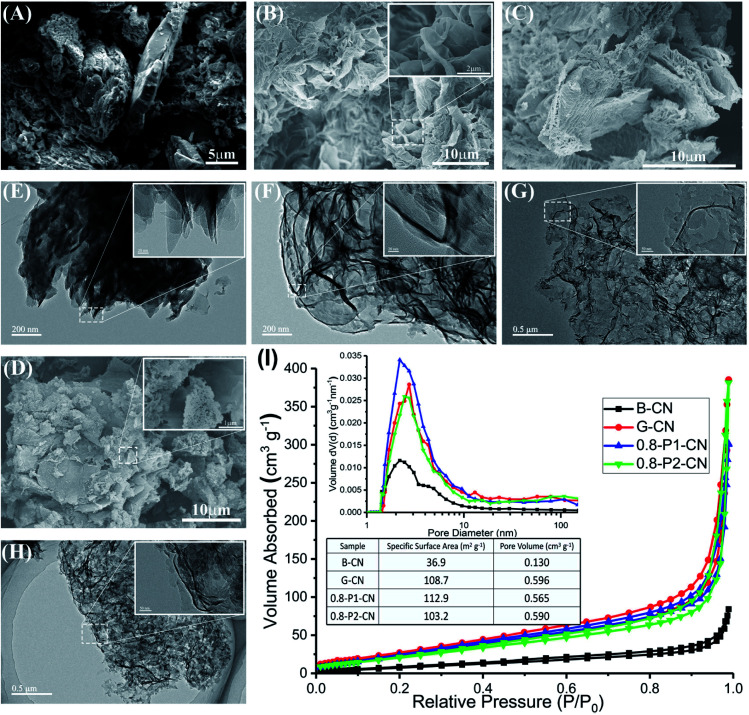
(A, B, C and D) SEM images of B-CN, G-CN, 0.8-P1-CN and 0.8-P2-CN, respectively. (E, F, G and H) TEM images of B-CN, G-CN, 0.8-P1-CN and 0.8-P2-CN, respectively. (I) N_2_ physisorption measurement results.

Fig. 1A–D show representative g-C_3_N_4_ morphologies characterized by field emission electron scanning microscope (SEM). Compared with the B-CN powder which shows a large agglomerate appearance (Fig. 1A), the g-C_3_N_4_ synthesized using the NH_4_Cl-assisted “bottom-up” strategy shows characteristics of thin nanosheets with crinkly structures (Fig. 1B–D). It can be seen that G-CN is mainly consisted of micrometer scale petals-like nanosheets with relatively smooth surfaces and edges (Fig. 1B). The 0.8-P1-CN nanosheets (Fig. 1C) remain a flower-like structure and part of the nanosheets become much smaller with serrated edges, which could be ascribed to the reaction of g-C_3_N_4_ nanosheets with H_3_PO_4_.^18^ In contrast, few flower-like structures are found in the H_3_PO_2_ doped powder (Fig. 1D) and even smaller nanosheets can be observed with some large nanosheets surrounded. Further to transmission electron microscope (TEM) images, the dense stacking structure of bulk B-CN is illustrated in Fig. 1E, while the as prepared g-C_3_N_4_ nanosheets using NH_4_Cl as dynamic gas template (Fig. 1F–H) present porous structures. In the N_2_ physisorption measurements, the specific surface areas, pore volumes and pore size distributions of the g-C_3_N_4_ samples were obtained (Fig. 1I). All the tested g-C_3_N_4_ samples possess well-defined mesopores. The specific areas and pore volumes of g-C_3_N_4_ nanosheets are nearly 3 and 4.5 times enlarged than those of B-CN, respectively. Thus, the porous g-C_3_N_4_ nanosheets assemblages appeared to us to provide more active sites for an enhanced photocatalytic performance.

### Structure characterization

**Fig. 2:**
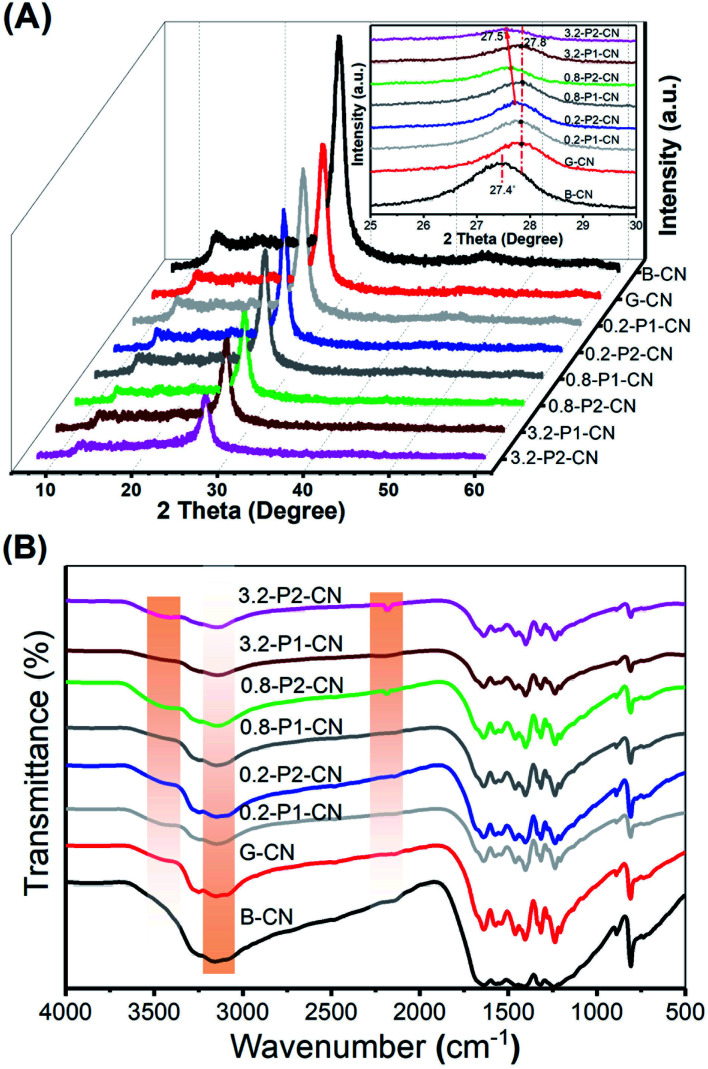
(A) XRD patterns of representative g-C_3_N_4_ samples. (B) FTIR spectra of these same samples.

In Fig. 2A we present the X-ray diffraction (XRD) patterns of the g-C_3_N_4_ samples. All samples show the two characteristic diffraction peaks at around 13.0° and 27.4°, which were assigned to the in-plane (100) and interlayer-stacking (002) crystal planes of g-C_3_N_4_, respectively.^2^ Compared with the peaks in B-CN, diffraction peaks of g-C_3_N_4_ nanosheets samples (G-CN and *M*-P*n*-CN) became weaker and the peak intensities of the phosphorus doped g-C_3_N_4_ nanosheets were continuously weakened with increasing addition of phosphorus dopants. Notably, all H_3_PO_2_ doped g-C_3_N_4_ samples shows lower peak intensities compared to the corresponding H_3_PO_4_ doped g-C_3_N_4_ nanosheets at the same amount of phosphorus usage, which indicates H_3_PO_2_ could more easily cause the loss of ordered structures within g-C_3_N_4_ framework.^2^ The magnified XRD patterns (inset in Fig. 2A) depict that the (002) diffraction peak of G-CN nanosheets shifted to 27.8°, suggesting a smaller gallery distance between the basic layers in the nanosheets. Earlier pioneering work^6,21^ demonstrated that so-called planarizing the potentially undulated layers in g-C_3_N_4_ would result in denser stacking, thereby resulting in shifts in the characteristic (002) diffraction peaks to higher 2*θ* angles. Thus, the (002) diffraction peaks shifts in XRD patterns also indicate the formation of planarized g-C_3_N_4_ nanosheets as also demonstrated in our SEM studies. Interestingly, the (002) peaks of the H_3_PO_2_ doped g-C_3_N_4_ nanosheets continuously shifted back to around 27.5° with increasing dopant addition, while those of the H_3_PO_4_ doped samples were nearly centered at the same position with G-CN (ESI, Fig. S2†). This (002) peak shift in the H_3_PO_2_ doped nanosheets should be attributed to the increased disorder in the g-C_3_N_4_ in-planar matrix and consequently resulted in larger layer distance compared with G-CN and H_3_PO_4_ doped g-C_3_N_4_ nanosheets.^1,2^ Thus, this interesting observation suggests that this H_3_PO_2_ doping protocol could help to relieve the layer stacking problems with remaining nanosheets structures.

In the Fourier Transform Infrared (FTIR) spectroscopy (Fig. 2B), the typical peaks of g-C_3_N_4_ at 810 cm^−1^ and in the range of 900–1800 cm^−1^ were observed among all the tested g-C_3_N_4_ samples, which originated from the heptazine ring out-of-plane bending and the N–C=N heterorings in the “melon” framework.^2^ Compared with the pristine B-CN, three distinct changes (highlighted by the orange shaded regions) occurred with varying dopants and their usages. One was the progressive loss of peak intensities in the phosphorus doped nanosheets located between 3000 and 3300 cm^−1^ which were attributed to the N–H stretching vibrations. Another difference observed at 2184 cm^−1^ as a new peak appeared in H_3_PO_2_ doped g-C_3_N_4_ nanosheets representing the stretching vibration mode of cyano groups (–C≡N).^2,22,23^ Moreover, a weak and broad peak centered at around 3460 cm^−1^ was developed in the g-C_3_N_4_ nanosheets samples and this peak could be assigned to the stretching vibration mode of hydroxyl groups (–OH).^24,25^ It is noteworthy that the hydroxyl group stretching vibration peaks in the H_3_PO_2_ doped nanosheets are relatively stronger than those in the H_3_PO_4_ doped samples, suggesting more exposure of hydroxyl groups in the H_3_PO_2_ doped g-C_3_N_4_ nanosheets, which will be further evidenced by the after-mentioned X-ray photoelectron spectroscopy analysis.

The FTIR results reveal that the addition of H_3_PO_2_ and H_3_PO_4_ in g-C_3_N_4_ nanosheets synthesis could significantly decrease N–H concentration and cyano groups can be very effectively introduced by using H_3_PO_2_. The introduction of oxygen containing groups like hydroxyl group should be attributed to the high temperature (550 °C) synthesis and the atmospheric sample storage. Notably, recent research results claimed that the photocatalytic activity of g-C_3_N_4_ can be greatly enhanced by introducing cyano groups into the melon framework.^2,24^ Thus, the H_3_PO_2_ doped g-C_3_N_4_ nanosheets are expected to possess superior photocatalytic activities as would be demonstrated in the H_2_ production test.

**Fig. 3:**
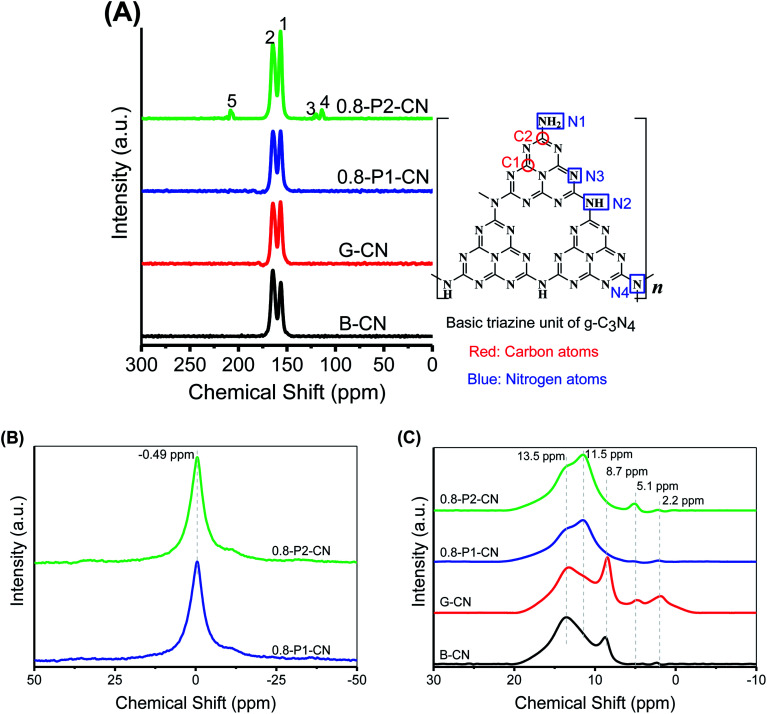
(A) Solid-state NMR spectra of ^13^C and basic g-C_3_N_4_ triazine unit structure. (B and C) Solid-state ^31^P and ^1^H NMR spectrums, respectively.

Besides XRD and FTIR studies, the chemical structures of the g-C_3_N_4_ samples were further characterized by solid-state ^13^C, ^31^P and ^1^H magic angle spinning (MAS) NMR spectra. Fig. 3A presents the ^13^C NMR spectra, in which all samples show two strong peaks at 156.3 and 164.6 ppm corresponding to the chemical shifts of C_3N_ (1) and C_2N–NH_*x*__ (2) in the corrugated g-C_3_N_4_ melon networks, respectively.^26–28^ Compared with B-CN, the C_3N_ (1) peak in the g-C_3_N_4_ nanosheets powders was intensified, which indicates the loss of NH_*x*_ groups, in accordance with the FTIR results. Moreover, G-CN and 0.8-P1-CN were found very similar, suggesting that the nonoxidative H_3_PO_4_ would not strongly disintegrate the g-C_3_N_4_ heptazine units. However, the C_3N_ (1) peak was further intensified in the 0.8-P2-CN sample and three new peaks (peak 3, 4 and 5) emerged. These new peaks with a chemical shift of 119.9, 112.3 and 208.1 ppm could be assigned to the carbon atoms in cyano groups, sp^2^ hybridized carbon atoms and the carbon atoms in the carbonyl group (–C=O) containing species, respectively.^2,28–30^

No obvious differences are observed on the ^31^P NMR spectra (Fig. 3B) of 0.8-P2-CN and 0.8-P1-CN nanosheets. Both samples show single sharp peak centered at −0.49 ppm which corresponds to the P–N coordinate bonds with phosphorus atom being connected to two adjacent pyridinic N atoms from two separated g-C_3_N_4_ triazine units,^31^ indicating that the phosphorus atoms sourced from H_3_PO_2_ and H_3_PO_4_ were in the same final state and interstitially doped in the g-C_3_N_4_ matrices.^32^ As the synthesis was conducted in the high temperature air, these doped phosphorus atoms would most likely be in pentavalent oxidation state and absorb moisture from air and eventually transform into the form of phosphoric acid. In terms of the reasons for the differences between the H_3_PO_4_ and H_3_PO_2_ doped g-C_3_N_4_ nanosheets, a detailed discussion will be presented later.

Considering NH_4_Cl and H_3_PO_2_ would generate HCl and H_3_PO_4_ which were commonly used for g-C_3_N_4_ protonation,^14,18^ the as prepared g-C_3_N_4_ could be protonated. As seen in Fig. 3C, the five peaks at 2.2, 5.1, 8.7, 11.5 and 13.5 ppm in the ^1^H NMR spectra were assigned to the protons in aliphatic groups, residual water, NH_*x*_ groups, the hydrogen bonds in acid groups (such as carboxylic acid and phosphoric acid) and g-C_3_N_4_ framework,^33–35^ respectively. Thus, the intensification of the 8.7 or 11.5 ppm peak can be regarded as evidence for the protonation of g-C_3_N_4_ nanosheets. Compared with B-CN, the 8.7 ppm peak in G-CN was obviously strengthened because of the formation of hydrogen bonds between the proton and nitrogen atoms in the g-C_3_N_4_ framework. Despite of the weakened peaks at 8.7 ppm over the phosphorus doped nanosheets, the intensified peaks at 11.5 ppm in 0.8-P1-CN and 0.8-P2-CN should be attributed to the protonation with protons connected with phosphoric acid groups.

The measured zeta potentials (ESI, Fig. S3†) reveal that the surface charge properties were significantly changed in the g-C_3_N_4_ nanosheets. The zeta potentials of G-CN, 0.8-P1-CN and 0.8-P2-CN were increased from −16.3 mV (B-CN) to −6.1, −3.1 and −4.5 mV, respectively, and these zeta potential changes further confirm the protonation of the g-C_3_N_4_ nanosheets.^15^ Besides the positive potential on the dispersibility, electronic bandgap structure and surface area of g-C_3_N_4_, one of the other important advantages of protonation is to induce higher ionic conductivity to the g-C_3_N_4_ framework and then enable the acceleration of charge carrier migration, which would benefit for better photocatalytic performances.^14,18,36^ Thus, higher H_2_ evolution ability could be expected for the protonated g-C_3_N_4_ nanosheets.

In addition, combining with the newly emerged carbonyl group peak in the ^13^C spectra and the enhanced acid groups hydrogen bonds ^1^H NMR signal in 0.8-P2-CN nanosheets, it is reasonable to propose that carboxyl groups (–COOH) were introduced into the H_3_PO_2_ doped nanosheets, thereby resulting in a stronger 11.5 ppm peak in 0.8-P2-CN than that of 0.8-P1-CN, and this peak intensification is in agreement with the observation that the H_3_PO_2_ doped g-C_3_N_4_ nanosheets showed stronger hydroxyl group (–OH) peak intensities in FTIR spectra.

### Chemical compositions

The g-C_3_N_4_ compositions obtained from organic elemental analysis (OEA) and X-ray photoelectron spectroscopy (XPS) can be found in Table 1 (ESI, XPS spectra in Fig. S4†). As can be seen in Table 1, the increased H/C ratios in g-C_3_N_4_ nanosheets can also be regarded as evidences for protonation.^15^ The N/C atomic ratio of B-CN obtained from OEA and XPS were 1.467 and 1.318, respectively. According to XPS results, the N/C ratio of G-CN slightly decreased from 1.318 to 1.283 when B-CN was transformed into G-CN, and this was ascribed to higher degree polymerization with more NH_3_ release during the formation of nanosheet structures.^7^ Although the N/C ratio of H_3_PO_4_ doped nanosheets dropped to about 1.249, it was still very close to that of G-CN. However, the N/C ratio of H_3_PO_2_ doped g-C_3_N_4_ nanosheets fell to 0.904 as a result of the intensive loss of nitrogen caused by the addition of H_3_PO_2_. Meanwhile, the O/C ratio of the H_3_PO_2_ doped g-C_3_N_4_ nanosheets drastically increased to 0.279. Thus, it was most likely that the H_3_PO_2_ induced the opening of g-C_3_N_4_ heptazine rings and caused nitrogen vacancies. The exposed defective edges would then react with oxygen to form oxygen containing groups such as carboxyl groups *etc.*^24^

**Table 1:** Element atomic ratios of the g-C_3_N_4_ samples^a^

Sample	OEA			XPS		
	N/C	O/C	H/C	N/C	O/C	P/C
B-CN	1.467	0.007	0.023	1.318	0.038	0
G-CN	1.421	0.018	0.027	1.283	0.064	0
0.2-P1-CN	1.389	0.047	0.029	1.287	0.079	0.018
0.8-P1-CN	1.381	0.064	0.031	1.266	0.105	0.038
3.2-P1-CN	1.365	0.073	0.032	1.249	0.112	0.043
0.2-P2-CN	1.293	0.091	0.030	1.204	0.127	0.010
0.8-P2-CN	1.117	0.135	0.035	1.037	0.223	0.017
3.2-P2-CN	1.002	0.162	0.037	0.904	0.279	0.024

a OEA and XPS represent the results obtained from organic elemental analysis and X-ray photoelectron spectroscopy, respectively.

In accordance with the fact that H_3_PO_2_ will decompose to release both PH_3_ and H_3_PO_4_,^4^ the differences highlighted between the H_3_PO_2_ and H_3_PO_4_ doped g-C_3_N_4_ nanosheets provides strong evidence that the introduction of nitrogen vacancies can be attributed to the strongly reductive PH_3_ rather than H_3_PO_4_. In the reduction synthesis of nitrogen defective g-C_3_N_4_,^16^ the energy changes for removing a lattice nitrogen atom located at N1, N2, N3 and N4 (referring to Fig. 3A) and terminating the dangling bonds of C atoms with H atoms using H_2_ as reduction agent were 0.65, 0.83, 1.40 and 2.39 eV, respectively,^16^ and the H_2_ reduction caused homogeneous sp^2^ hybridized nitrogen atoms losses on the g-C_3_N_4_ heptazine rings. Changing H_2_ to PH_3_, we calculated the corresponding energy changes and the four energy changes decrease to 0.61, 0.75, 1.28 and 2.27 eV (ESI†), respectively, indicating the stronger reduction agent PH_3_ would be more favorable for opening heptazine rings and result in significant nitrogen atoms *in situ* reduction removal. Thus, it was reasonable to propose that intensive nitrogen vacancies could be introduced into the H_3_PO_2_ doped g-C_3_N_4_ nanosheets because of that the strong reducing agent PH_3_, produced *via* H_3_PO_2_ decomposition.

**Fig. 4:**
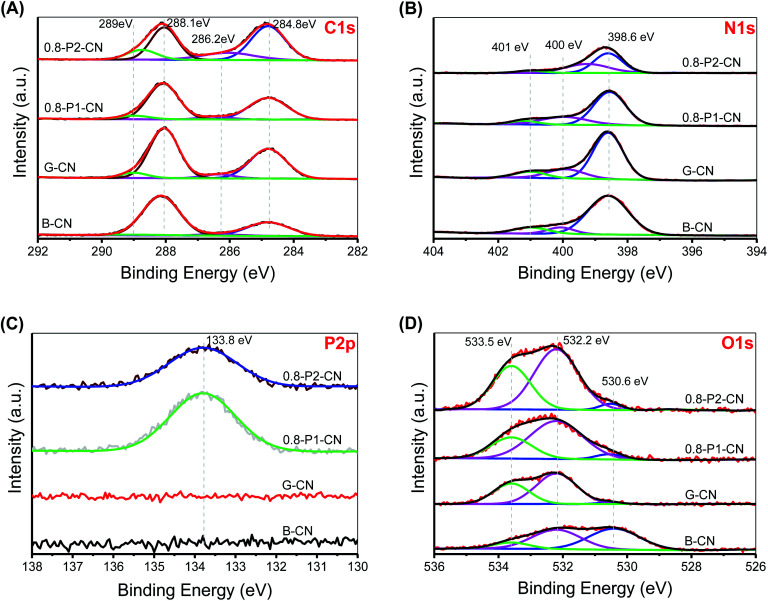
(A) C 1s XPS narrow scan. (B) N 1s XPS narrow scan. (C) P 2p XPS narrow scan. (D) O 1s XPS narrow scan.

The narrow scan C 1s, N 1s, P 2p and O 1s XPS spectra were collected and deconvoluted into their components (Fig. 4A–D). The four peaks in C 1s spectra are located at 284.8, 286.2, 288.1 and 289 eV, corresponding to the adventitious hydrocarbons (C–C or C=C), C–NH_*x*_ (*x* = 1, 2), N–C=N coordination and carboxyl groups, respectively.^2,37,38^ It is obvious that the 288.1 eV signal lost intensity, while the 286.2 eV peak is significantly intensified in the 0.8-P2-CN powder. As cyano group has similar C 1s binding energy to C-NH_*x*_,^2^ the intensified 286.2 eV signal together with the weakened N–C=N coordination peak could be further evidence for the heptazine rings opening and the formation of cyano groups in the H_3_PO_2_ doped nanosheets. Besides, the enhanced 289 eV peak in 0.8-P2-CN confirms the considerable introduction of carboxyl groups into H_3_PO_2_ doped g-C_3_N_4_ nanosheets. The three peaks centered at 398.6, 400 and 401 eV in the N 1s XPS spectra are assigned to the sp^2^-hybridized (N_2C_), sp^3^-hybridized (N_3C_) nitrogen atoms and NH_*x*_ groups, respectively.^2^ For the H_3_PO_2_ doped nanosheets, the drastically weakened NH_*x*_ peak and shift of N_3C_ to lower binding energy also indicate the NH_*x*_ loss and formation of cyano groups, which are in agreement with the results obtained by FTIR and NMR spectra.

Both the P 2p XPS spectra of the H_3_PO_4_ and H_3_PO_2_ doped nanosheets show one single pentavalent phosphorus peak centered at 133.8 eV, indicating that no P–C bonds formed.^18^ Moreover, the elemental mapping conducted on scanning transmission microscope (ESI, Fig. S5†) reveal that phosphorus is distributed evenly in both 0.8-P1-CN and 0.8-P2-CN with an actual content of around 0.5 wt%. The parallel intensification of the 533.5 and 532.2 eV peaks representing –OH and C=O groups^39–41^ in the 0.8-P2-CN O 1s spectra are consistent with the hypothesis that carboxyl groups was introduced into the H_3_PO_2_ doped g-C_3_N_4_ nanosheets. According to the summarized XPS data (ESI, Table S1†), the N_2C_/C ratio in the g-C_3_N_4_ nanosheets remarkably dropped from 0.917 to 0.568 and the corresponding O/C ratio increased from 0.062 to 0.217 with the addition of H_3_PO_2_, these results also suggest the introduction of nitrogen defects and oxygen species into the H_3_PO_2_ doped g-C_3_N_4_ nanosheets.

### Proposed schematic structures

**Fig. 5:**
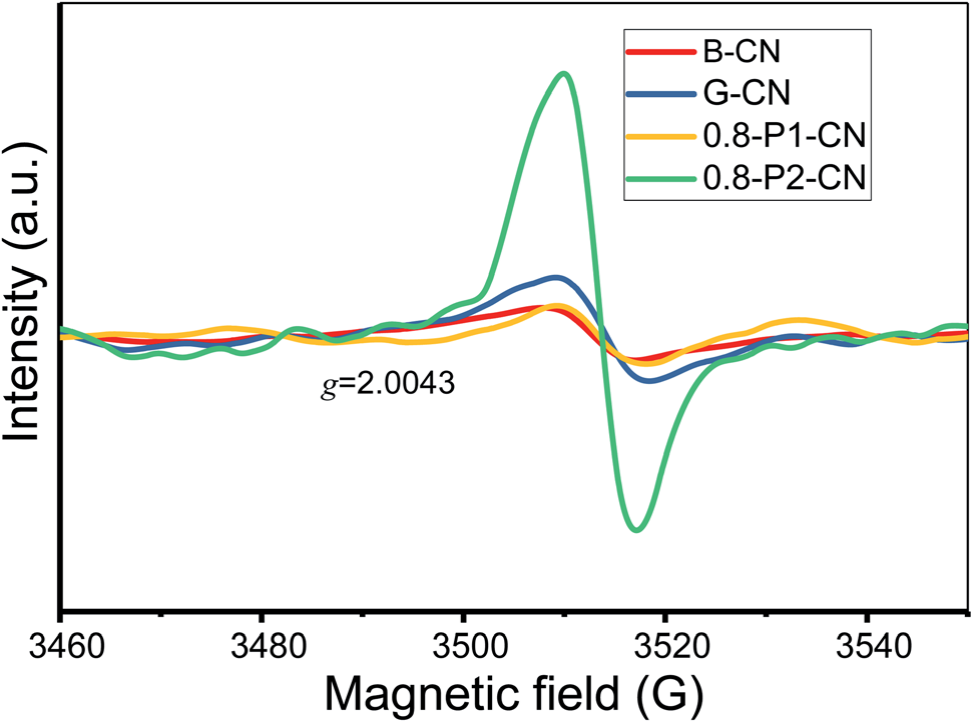
EPR spectra of the representative g-C_3_N_4_ samples.

Further to our XPS and OEA analysis, electron paramagnetic resonance (EPR) experiments were conducted to provide fingerprint evidence for probing the surface nitrogen vacancies introduced to the g-C_3_N_4_ nanosheets. Several different samples having closely similar weights were examined to ensure a meaningful comparison of the peak intensities (areas). As shown in Fig. 5, all samples display a single Lorentzian line with an electronic *g* value of 2.0043 in the magnetic field from 3460 to 3560 G, which represents the unpaired electrons of sp^2^ hybrid carbon atoms in π-conjugated aromatic rings.^42,43^ Thus the formation of two-coordinated nitrogen vacancies in the heptazine rings of g-C_3_N_4_ would donate unpaired electrons to the sp^2^-carbon atoms. Therefore, compared with the negligible EPR signals of B-CN, G-CN and 0.8-P1-CN, the significantly enhanced EPR intensity in 0.8-P2-CN nanosheets adds weight to the idea of the opening of heptazine rings and the formation of two-coordinated nitrogen vacancies.

**Fig. 6:**
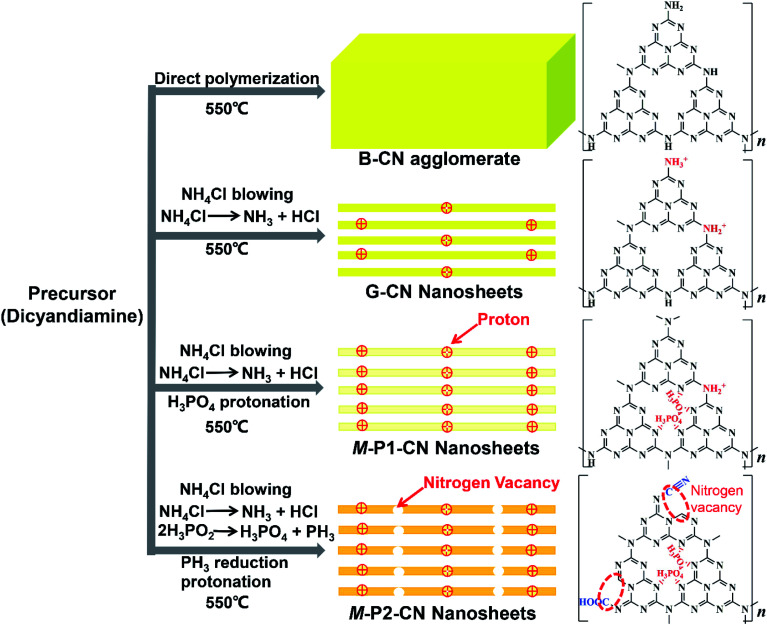
The g-C_3_N_4_ evolution process and proposed chemical structures.

Thus, we believe that combining the various techniques including FTIR, NMR, zeta potential, XPS and EPR analysis, the controllable generation of nitrogen vacancies, protonation and newly introduced functional groups in g-C_3_N_4_ framework are soundly confirmed. From all this information, the proposed schematic molecule structures and evolution processes of the g-C_3_N_4_ samples can now be advanced (Fig. 6).

In brief, compared with the directly polymerized bulk g-C_3_N_4_ agglomerate, protonated g-C_3_N_4_ nanosheets could be readily fabricated with the help of dynamic gas template NH_4_Cl. With co-addition of H_3_PO_4_ as dopant, the g-C_3_N_4_ nanosheets could be further protonated with phosphorus atoms interstitially doped into the frameworks. However, apart from the additional protonation, the addition of the reducing dopant H_3_PO_2_ could also induce significant nitrogen loss to generate nitrogen vacancies and variously introduce, cyano and carboxyl functional groups, into the g-C_3_N_4_ nanosheets.

### Optical properties

**Fig. 7:**
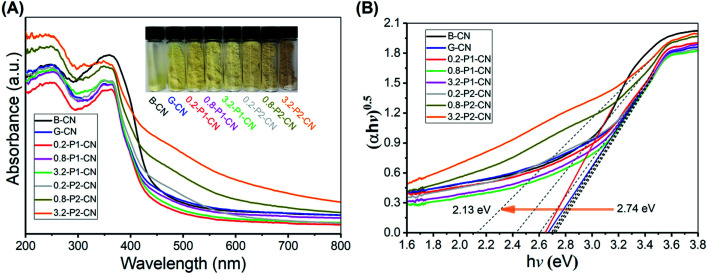
(A) UV-Vis DRS spectra and. (B) Plots of transformed Kubelka–Munk function *versus* proton energy for a variety of g-C_3_N_4_ samples.

The optical properties and light harvesting abilities of g-C_3_N_4_ samples were significantly modified by the specific addition of NH_4_Cl and phosphorus dopants. The obvious changes were well observed as the yellow dense B-CN powder was transformed into porous and fluffy states having different colours (Fig. 7A). Compared with B-CN and G-CN, the colours of the *M*-P1-CN nanosheets became lighter with the increasing addition of H_3_PO_4_, whereas those of the H_3_PO_2_ doped nanosheets turned from dark yellow to brown. The absorption edge of G-CN shifted to a lower wavelength as compared with B-CN and it continuously shifted to the lower direction with increasing H_3_PO_4_ addition. The blueshifts in the G-CN and H_3_PO_4_ doped nanosheets were attributed to the enhanced protonation.^5,14^ Unlike H_3_PO_4_, a progressive redshift was achieved by increasing H_3_PO_2_ usage, which indicates the bandgap structures of the H_3_PO_2_ doped nanosheets could be easily tuned by adjusting the amount of H_3_PO_2_.

According to the transformed Kubelka–Munk function determined electronic bandgaps shown in Fig. 7B, the bandgaps of H_3_PO_4_ doped g-C_3_N_4_ nanosheets were slightly increased to a narrow range of 2.70–2.74 eV from 2.67 eV (G-CN), while that of the H_3_PO_2_ doped nanosheets can be significantly narrowed to 2.13 eV. The narrowed bandgaps reveal the enhanced visible light harvesting ability of the nitrogen deficient g-C_3_N_4_ nanosheets which were doped by H_3_PO_2_.

To understand the influence of protonation (including both the protons which connect with nitrogen atoms and the interstitially doped phosphoric acid groups), nitrogen vacancies and carboxyl groups (–COOH) on the bandgaps of the g-C_3_N_4_ samples, partial density of states (PDOS) and density-functional theory (DFT) calculations were performed (ESI for details, Fig. S6†).

**Fig. 8:**
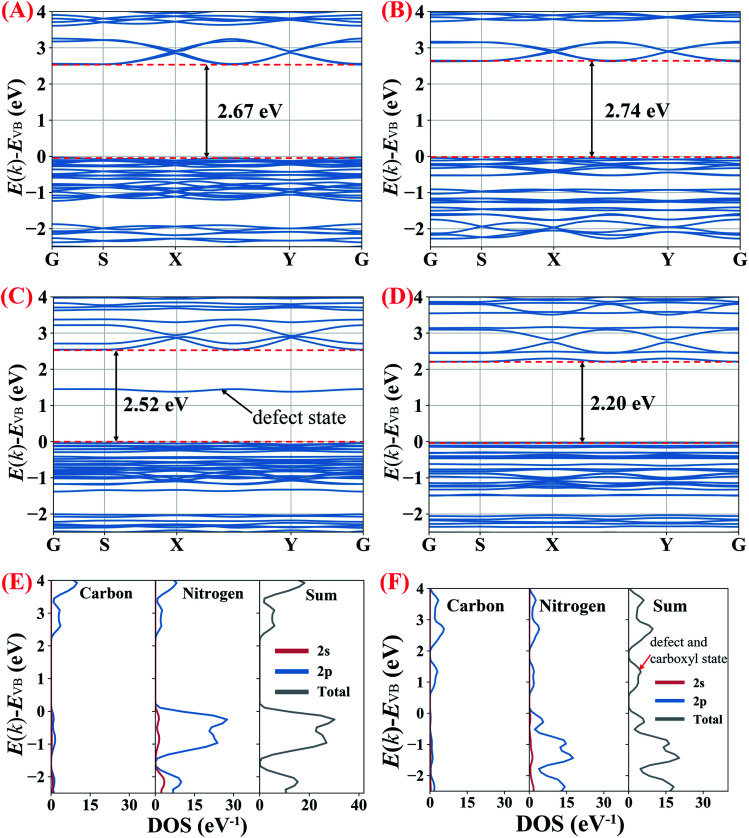
(A–D) Calculated band structures of B-CN and g-C_3_N_4_ with protonation, nitrogen vacancies and carboxyl groups, respectively. (E and F) corresponding PDOS for B-CN and g-C_3_N_4_ containing nitrogen vacancies and carboxyl groups, respectively.

As shown in Fig. 8A and B, the calculated bandgap for B-CN is 2.67 eV which becomes slightly enhanced to 2.74 eV in the protonated g-C_3_N_4_ nanosheets, indicating that the protonation (no matter induced by NH_4_Cl gas template or interstitially doped phosphorus) does not significantly impact on the magnitude of bandgap. To clarify the effects of the nitrogen vacancies and carboxyl groups, g-C_3_N_4_ unit cells only containing nitrogen vacancies or carboxyl groups were built, respectively. The calculations in Fig. 8C and D show that the nitrogen vacancies and the carboxyl groups decrease the bandgap to 2.52 and 2.20 eV, respectively. The defect energy level observed in the g-C_3_N_4_ only containing nitrogen vacancies, composing of both C 2p and N 2p orbitals, is about 1.5 eV above valence band (VB). The narrowed bandgap of the g-C_3_N_4_ containing nitrogen vacancies and carboxyl groups agrees with the trend observed in the UV-Vis DRS analysis above. As the PDOS seen in Fig. 8E, the conduction band (CB) of B-CN is composed of C 2p and N 2p orbits, while C 2p and N 2p orbits mainly contribute to valence band; this result is consistent with previous work.^2,44^ According to Fig. 8F, the significantly narrowed bandgap width of the H_3_PO_2_ doped g-C_3_N_4_ nanosheets is due primarily to a lowering of CB minimum by about 0.4 eV with the emergence of defect and carboxyl states. These results therefor confirm that the coexistence of nitrogen vacancies and carboxyl groups would decrease the width of g-C_3_N_4_ bandgap. As confirmed in previous work,^2^ the coexistence of cyano groups and nitrogen vacancies would also lower the CB minimum and result in narrower electronic energy bandgaps. By analogy, the coexistence of cyano groups, carboxyl groups and nitrogen vacancies would synergistically decrease the bandgap width of the as prepared g-C_3_N_4_ nanosheets which were doped by H_3_PO_2_.

**Fig. 9:**
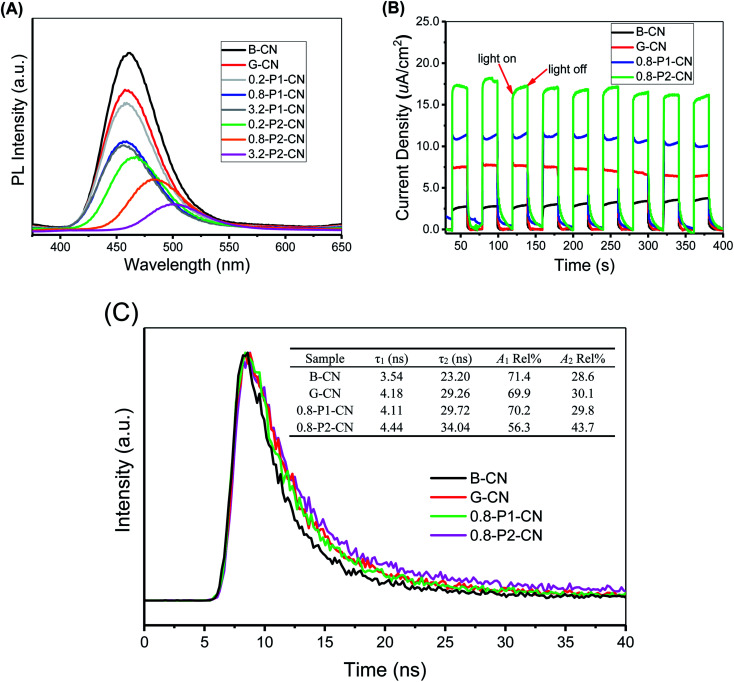
(A) Photoluminescence (PL) spectra and (B) photocurrent curves for B-CN and representative g-C_3_N_4_ nanosheet materials. (C) Time-resolved photoluminescence decay spectra for representative g-C_3_N_4_ samples.

As the photoluminescence (PL) spectra illustrated in Fig. 9A, B-CN shows an intense fluorescence signal at around 460 nm under visible-light irradiation and this signal in G-CN is significantly decreased. Because the PL spectra can be interpreted as the radiative recombination of surface trapping states, thus the weakened PL intensity could indicate the enhanced separation of photo-excited electrons and holes.^45,46^ By adding H_3_PO_4_, the peak is further weakened. It is noted that much lower peak intensities are found over H_3_PO_2_ doped g-C_3_N_4_ nanosheets and the peak continuously shifts to around 510 nm with the increasing addition of H_3_PO_2_. The PL peak red-shift in the H_3_PO_2_ doped g-C_3_N_4_ nanosheets is associated with the decreased bandgap energy of the samples and consistent with the trend as seen in UV-Vis DRS.^46^ As illustrated in Fig. 9B, all samples exhibit sensitive photocurrent responses during the visible-light on/off irradiation. The photocurrent density values for the four representative samples (B-CN, G-CN, 0.8-P1-CN and 0.8-P2-CN) are ≈2.5, 7.5, 11.1 and 17.0 μA cm^−2^, respectively.

To investigate the dynamic electron immigration process, time-resolved fluorescence spectra were collected. As seen in Fig. 9C, the lifetime of the photo-induced charge carriers in the H_3_PO_2_ doped g-C_3_N_4_ nanosheets were significantly prolonged compared with B-CN, G-CN and H_3_PO_4_ doped nanosheets sample, revealing the accelerated charge transfer performance.^46^

Both photoluminescence and photocurrent tests indicate that the g-C_3_N_4_ nanosheets possess much better performance in the critical process of photo-excited charge carrier separation and visible-light response than the corresponding directly polymerized B-CN. Regarding G-CN and H_3_PO_4_ doped g-C_3_N_4_ nanosheets, the enhanced separation of charge carriers could be attributed to their large specific surface areas and protonation.^7,14^ Besides, the interstitially doped phosphorus atoms in the H_3_PO_4_ doped g-C_3_N_4_ nanosheets would likely facilitate optimization of the π-conjugated heptazine rings to improve the carrier mobility and offer a new channel for carrier migration,^18,32^ thereby offering enhanced charge carriers separation. Besides the interstitial phosphorus doping, the nitrogen vacancies in the H_3_PO_2_ doped g-C_3_N_4_ nanosheets would also induce unpaired sp^2^-carbon atoms within the π-conjugated heptazine rings. These electron defected sp^2^-carbon atoms and newly introduced electron-withdrawing groups (cyano and carboxyl groups) can redistribute the π-electrons and result in improved visible light absorption and photo-excited charge carriers separation.^4,47–49^ Thus, a confluence of the synergistic effect of protonation, interstitial phosphorus doping, nitrogen vacancies and newly introduced functional groups in the H_3_PO_2_ doped g-C_3_N_4_ nanosheets was achieved and expected to offer a comprehensive enhancement of visible-light photocatalysis. We now turn to these experiments.

### Photocatalytic H_2_ evolution performances

**Fig. 10:**
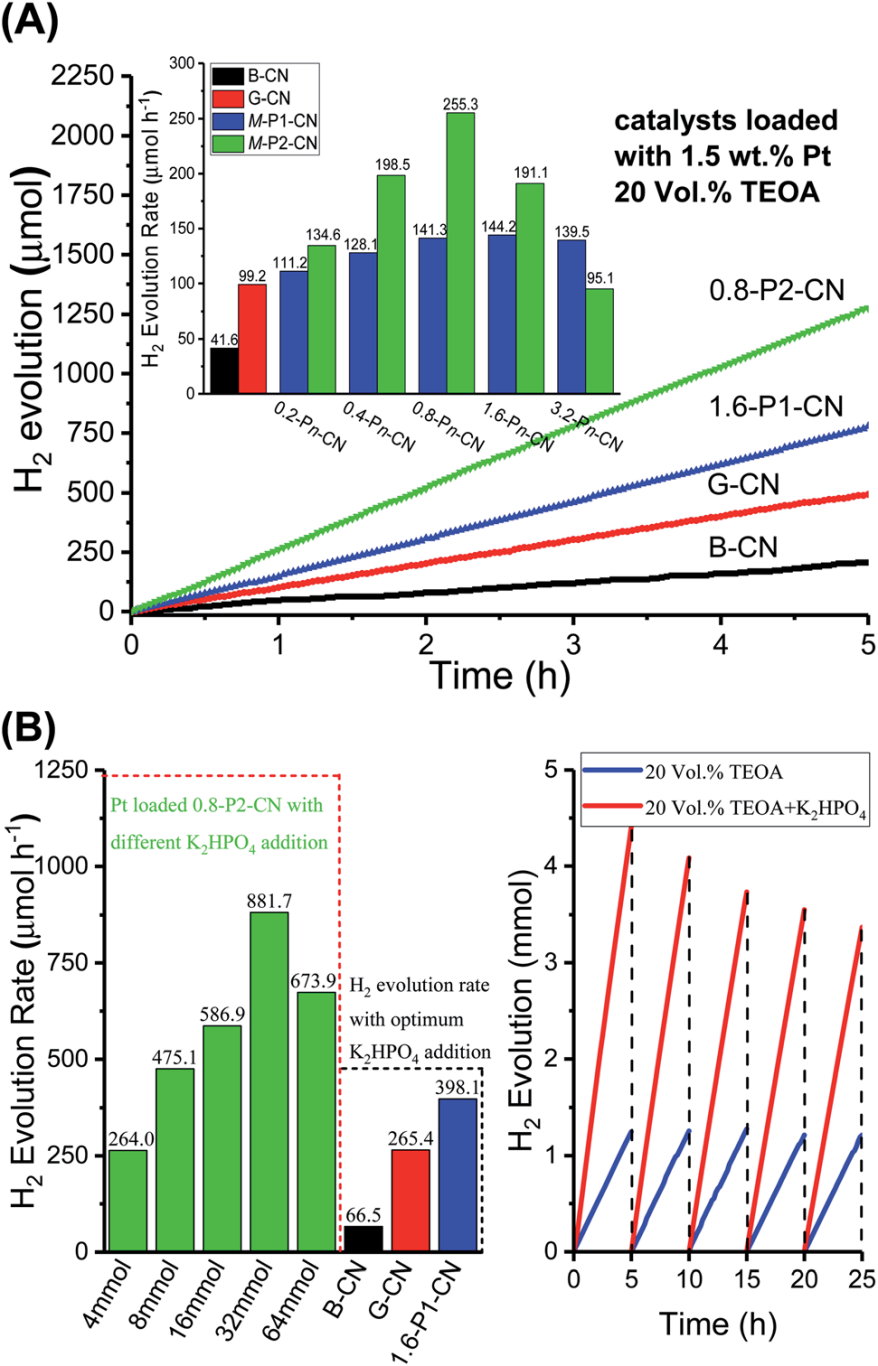
(A) Rates and time-on-stream of the photocatalytic H_2_ evolution over 1.5 wt% Pt-loaded g-C_3_N_4_ samples in 20 vol% TEOA solution under visible-light irradiation (*λ* ≥ 400 nm). The g-C_3_N_4_ usage in each experiment was 15 mg. (B) H_2_ evolution rates over the 1.5 wt% Pt-loaded B-CN, G-CN, 1.6-P1-CN and 0.8-P2-CN in the 20 vol% TEOA solution with optimum addition of K_2_HPO_4_ (left). Time course of the long-term H_2_ evolution over the 0.8-P2-CN nanosheets loaded with 1.5 wt% Pt in 20 vol% TEOA and the optimum TEOA/K_2_HPO_4_ mixture solution (right).

The photocatalytic performances of the g-C_3_N_4_ samples were evaluated by measuring the visible-light (*λ* ≥ 400 nm) H_2_ evolution in 20 vol% triethanolamine (TEOA) aqueous solution with 1.5 wt% of Pt loading. As shown in Fig. 10A, the g-C_3_N_4_ nanosheets exhibited much higher photocatalytic activities than the standard reference B-CN. The H_2_ generation rate of G-CN reached 99.1 μmol h^−1^, whilst only 41.6 μmol h^−1^ was obtained over B-CN. Importantly, the H_2_ evolution rate of g-C_3_N_4_ nanosheets was significantly improved with the doping of H_3_PO_4_ and H_3_PO_2_, respectively. The H_3_PO_4_ doped g-C_3_N_4_ nanosheets exhibited a H_2_ evolution rate of 144.2 μmol h^−1^ by the 1.6-P1-CN sample. Interestingly, 0.8-P1-CN and 3.2-P1-CN gave a very similar H_2_ evolution rate as 1.6-P1-CN, which could be ascribed to that the limited visible light absorption of the H_3_PO_4_ protonated g-C_3_N_4_ nanosheets confines the highest H_2_ evolution rate when a certain level of H_3_PO_4_ addition was reached. For the H_3_PO_2_ doped nanosheets, 0.8-P2-CN showed the highest H_2_ evolution rate of 255.3 μmol h^−1^ which was 6.14, 2.58 and 1.77 times of that over B-CN, G-CN and 1.6-P1-CN, respectively. The bandgap structures determined by the valence band XPS spectra and UV-Vis DRS results indicate that the narrowed bandgap of the H_3_PO_2_ doped g-C_3_N_4_ nanosheets originates from the conduction band decrease (ESI, Fig. S7 and Table S2†). Thus, the excessively lowered reduction driving force for H_2_ evolution may result in lower H_2_ evolution activity even though the visible light harvesting ability of g-C_3_N_4_ was progressively intensified by increasing H_3_PO_2_ usage. These findings could explain why the highest H_2_ generation performance was achieved on the Pt loaded 0.8-P2-CN.

Because our RTK-Solar H_2_ evolution system was used for the first time for photocatalytic H_2_ evolution tests, its accuracy was verified in two steps. First, the evolved gas was confirmed to be only H_2_ with the help of Hiden HPR 20 gas chromatograph/mass spectrometer system (ESI, Fig. S8†). Second, H_2_ evolution experiments over 1.5 wt% Pt loaded B-CN, G-CN, 1.6-P1-CN and 0.8-P2-CN in 20 vol% TEOA solution were further conducted under the same conditions as those in the RTK-Solar system. With the quartz reactor being connected to a gas circulation system, the generated H_2_ amount was quantified by a calibrated gas chromatography equipped TCD detector (Clarus 580, PerkinElmer, helium as carrier gas) for every 1 hour. The gas chromatography determined H_2_ evolution rates over the 1.5 wt% Pt loaded B-CN, G-CN, 1.6-P1-CN and 0.8-P2-CN were 42.8, 106.6, 140.1 and 261.6 μmol h^−1^, respectively (ESI, Fig. S9 and Table S3†). The ratios of the H_2_ evolution rates determined by gas chromatography method and the RTK-Solar system over the four representative g-C_3_N_4_ samples were located in a very narrow range of 0.972 to 1.076, confirming the reliability of RTK-Solar system for measuring the amount of generated H_2_. The photocatalytic H_2_ evolution results reveal the significantly enhanced g-C_3_N_4_ photocatalytic performance over the nitrogen deficient and protonated g-C_3_N_4_ nanosheets produced by this facile *in situ* reductive synthesis strategy.

As proposed by Ye,^1^ using the nature-inspired strategy of adding K_2_HPO_4_ to TEOA solution, the H_2_ evolution performance of g-C_3_N_4_ could be significantly enhanced. In Ye's important work,^1^ it was shown that the added HPO_4_^2−^ can function both as a proton-pump in natural photosynthesis (to facilitate proton transport in the reaction solution) and also act as a mediator to give rise to a new proton-reduction pathway. The use of HPO_4_^2−^ instead of H_2_O provides the necessary protons to react with photo-generated electrons to produce H_2_ and PO_4_^3−^ on the surface of Pt co-catalyst at the very beginning. Following the H_2_ evolution from HPO_4_^2−^, the resulting PO_4_^3−^ entity immediately combined with H^+^ from H_2_O to regenerate HPO_4_^2−^ and finally complete the proton-reduction cycle.

In other words, the added K_2_HPO_4_ would not be consumed during the photocatalytic H_2_ evolution. In addition, K_2_HPO_4_ would also promote the oxidation of TEOA. The synergy of enhanced proton reduction and improved photooxidation of TEOA boosted the visible-light photocatalytic H_2_ evolution over the Pt loaded g-C_3_N_4_ catalysts. Thus, enhanced H_2_ evolution over the Pt loaded 0.8-P2-CN could also be expected by adding K_2_HPO_4_ to the TEOA solution.

As illustrated in Fig. 10B, with the optimum addition of K_2_HPO_4_ (32 mmol) to the 20 vol% TEOA solution, the H_2_ evolution of the Pt loaded 0.8-P2-CN was drastically enhanced to a rate of 881.7 μmol h^−1^, and impressive H_2_ generation could be observed (ESI, Video S1†). Similarly, the H_2_ generation rates of the Pt loaded B-CN, G-CN and 1.6-P1-CN were also promoted by the optimum TEOA/K_2_HPO_4_ solution, but the 0.8-P2-CN with 1.5 wt% Pt loading showed the highest increase among others. Though, G-CN and 1.6-P1-CN nanosheets possess well improved charge carrier separation abilities through protonation and phosphorus interstitial doping, and their H_2_ evolution rates could greatly enhanced by adding K_2_HPO_4_ to facilitate both proton reduction and the photooxidation of TEOA. However, their limited visible light absorption abilities with a wide bandgap of around 2.7 eV will confine the further improvement of visible-light H_2_ evolution.

In contrast, the 0.8-P2-CN nanosheets could harvest much more visible light to generate photo-excited electrons and holes with a significantly narrowed bandgap of 2.42 eV (ESI, Fig. S7†). Thus, together with the enhanced charge carrier separation, proton reduction and photooxidation of TEOA, the significantly intensified visible-light absorption ability could help the Pt loaded 0.8-P2-CN nanosheets to boost visible-light H_2_ evolution rate to an extremely high level.

In our 25 hours long-term H_2_ evolution experiments, it was found that the Pt loaded 0.8-P2-CN generated nearly the same amount of H_2_ for each 5 hours period in the TEOA solution, while the H_2_ generated in the last 5 hours period could still remain a 76.5% amount of that in the first 5 hours for the optimized TEOA/K_2_HPO_4_ mixture solution. As stated above, the K_2_HPO_4_ would not be consumed during the photocatalytic H_2_ evolution. The gradual decrease of the long-term H_2_ evolution rate in the TEOA/K_2_HPO_4_ solution should be attributed to the significant consumption of TEOA during the photocatalytic H_2_ evolution process. Thus, both the long-term H_2_ evolution experiments conducted in TEOA and TEOA/K_2_HPO_4_ solutions indicated the excellent photocatalytic stability of the H_3_PO_2_ doped nanosheets. The FTIR, elemental analysis conducted using STEM and XPS narrow scan results of the fresh and used 0.8-P2-CN suggest that there are no obvious changes which may induce adverse effect on the photocatalytic performances, confirming the stability of the as-prepared g-C_3_N_4_ nanosheets (ESI, Fig. S10–S12†).

In addition, the spent 0.8-P2-CN catalyst still showed observable H_2_ generation ability (ESI, Video S2†). The 0.8-P2-CN with 1.5 wt% Pt loading achieved an AQY of 10.7% and 40.4% at 420 nm in the TEOA and TEOA/K_2_HPO_4_ (ESI, Fig. S13†), respectively, indicating the extremely efficient visible-light photocatalytic H_2_ evolution over the H_3_PO_2_ doped g-C_3_N_4_ nanosheets compared with the previously reported work (Table S4†). The AQY measurement results at other wavelengths including 400, 450, 500, 550 and 600 nm were also supplemented in the ESI (Fig. S14 and Table S5†).

## Conclusions

Nitrogen deficient and protonated g-C_3_N_4_ nanosheets were successfully synthesized through a one-step, *in situ* reduction process combining both a NH_4_Cl-assisted strategy and H_3_PO_2_ doping. It has been demonstrated that the protonated g-C_3_N_4_ nanosheets have superb photocatalytic activities and can be robustly fabricated by using NH_4_Cl as a dynamic gas template. Compared with the g-C_3_N_4_ nanosheets which were simply further protonated by interstitial H_3_PO_4_ doping, the electronic bandgaps of the nitrogen deficient g-C_3_N_4_ nanosheets can be easily controlled by adjusting the added H_3_PO_2_. The enhanced protonation and reduced electronic bandgaps of H_3_PO_2_ doped g-C_3_N_4_ nanosheets can not only readily harvest visible light but also serve to separate the excited state charge carriers more effectively, thereby offering extremely efficient H_2_ production under visible-light irradiation.

With the addition of K_2_HPO_4_ to TEOA solution, the H_2_ evolution rate over the 1.5 wt% Pt loaded H_3_PO_2_ doped g-C_3_N_4_ nanosheets could be boosted to 881.7 μmol h^−1^ with an apparent quantum yield of 40.4% at 420 nm. In addition, this comprehensive investigation on different phosphorus compounds for g-C_3_N_4_ nanosheets modification indicates that H_3_PO_2_ is a promising dopant to synergistically modify the g-C_3_N_4_ photocatalysts in a single step. We believe this work will provide guidance for the facile synthesis of nitrogen defective and protonated g-C_3_N_4_-based materials for further synergistic enhancements of visible-light photocatalysts performance.

## Conflicts of interest

There are no conflicts to declare.

## Supplementary Material

SC-011-C9SC05060D-s001

## References

[cit1] Liu G., Wang T., Zhang H., Meng X., Hao D., Chang K., Li P., Kako T., Ye J. (2015). Angew. Chem..

[cit2] Yu H., Shi R., Zhao Y., Bian T., Zhao Y., Zhou C., Waterhouse G. I. N., Wu L., Tung C., Zhang T. (2017). Adv. Mater..

[cit3] Ge L., Han C., Liu J. (2011). Appl. Catal., B.

[cit4] Liu X., Wang P., Zhai H., Zhang Q., Huang B., Wang Z., Liu Y., Dai Y., Qin X., Zhang X. (2018). Appl. Catal., B.

[cit5] Zhang G., Zang S., Lin L., Lan Z. A., Li G., Wang X. (2016). ACS Appl. Mater. Interfaces.

[cit6] Niu P., Zhang L., Liu G., Cheng H. M. (2012). Adv. Funct. Mater..

[cit7] Lu X., Xu K., Chen P., Jia K., Liu S., Wu C. (2014). J. Mater. Chem. A.

[cit8] Li C., Yu S., Dong H., Liu C., Wu H., Che H., Chen G. (2018). Appl. Catal., B.

[cit9] Li C., Du Y., Wang D., Yin S., Tu W., Chen Z., Kraft M., Chen G., Xu R. (2017). Adv. Funct. Mater..

[cit10] Li C., Xu Y., Tu W., Chen G., Xu R. (2017). Green Chem..

[cit11] Li Z., Kong C., Lu G. (2015). J. Phys. Chem. C.

[cit12] Zhu Y. P., Ren T. Z., Yuan Z. Y. (2015). ACS Appl. Mater. Interfaces.

[cit13] Niu P., Liu G., Cheng H. M. (2012). J. Phys. Chem. C.

[cit14] Zhang Y., Thomas A., Antonietti M., Wang X. (2008). J. Am. Chem. Soc..

[cit15] Wu M., Ding T., Cai J., Wang Y., Xian H., Zhang H., Tian Y., Zhang T., Li X. (2018). ACS Sustainable Chem. Eng..

[cit16] Niu P., Yin L. C., Yang Y. Q., Liu G., Cheng H. M. (2014). Adv. Mater..

[cit17] Hong Z., Shen B., Chen Y., Lin B., Gao B. (2013). J. Mater. Chem. A.

[cit18] Shi L., Chang K., Zhang H., Hai X., Yang L., Wang T., Ye J. (2016). Small.

[cit19] Zhang Y., Mori T., Ye J., Antonietti M. (2010). J. Am. Chem. Soc..

[cit20] Yuan J., Gao Q., Li X., Liu Y., Fang Y., Yang S., Peng F., Zhou X. (2014). RSC Adv..

[cit21] Groenewolt M., Antonietti M. (2005). Adv. Mater..

[cit22] Cui Y., Ding Z., Fu X., Wang X. (2012). Angew. Chem..

[cit23] Lei W., Portehault D., Dimova R., Antonietti M. (2011). J. Am. Chem. Soc..

[cit24] Fu J., Zhu B., Jiang C., Cheng B., You W., Yu J. (2017). Small.

[cit25] Dong G., Ai Z., Zhang L. (2014). RSC Adv..

[cit26] Sehnert J., Baerwinkel K., Senker J. (2007). J. Phys. Chem. B.

[cit27] Jürgens B., Irran E., Senker J., Kroll P., Müller H., Wolfgan S. (2003). J. Am. Chem. Soc..

[cit28] Lau V. W. H., Moudrakovski I., Botari T., Weinberger S., Mesch M. B., Duppel V., Senker J., Blum V., Lotsch B. V. (2016). Nat. Commun..

[cit29] Fu Y., Zhu J., Hu C., Wu X., Wang X. (2014). Nanoscale.

[cit30] Makowski S. J., Gunzelmann D., Senker J., Schnick W. (2009). Z. Anorg. Allg. Chem..

[cit31] Zhu M., Kim S., Mao L., Fujitsuka M., Zhang J., Wang X., Majima T. (2017). J. Am. Chem. Soc..

[cit32] Ma X., Lv Y., Xu J., Liu Y., Zhang R., Zhu Y. (2012). J. Phys. Chem. C.

[cit33] Goward G. R., Schuster M. F., Sebastiani D., Schnell I., Spiess H. W. (2002). J. Phys. Chem. B.

[cit34] Emmler T., Gieschler S., Limbach H. H., Buntkowsky G. (2004). J. Mol. Struct..

[cit35] Chen Y., Lin B., Wang H., Yang Y., Zhu H., Yu W., Basset J. M. (2016). Chem. Eng. J..

[cit36] Ye C., Li J. X., Li Z. J., Li X. B., Fan X. B., Zhang L. P., Chen B., Tung C. H., Wu L. Z. (2015). ACS Catal..

[cit37] Rong M., Cai Z., Xie L., Lin C., Song X., Luo F., Wang Y., Chen X. (2016). Chem.–Eur. J..

[cit38] Gao H., Yan S., Wang J., Huang Y. A., Wang P., Li Z., Zou Z. (2013). Phys. Chem. Chem. Phys..

[cit39] Yatsimirskii K. B., Nemoskalenko V. V., Aleshin V. G., Bratushko Y. I., Moiseenko E. P. (1977). Chem. Phys. Lett..

[cit40] Mohamed M. A., Zain M. F. M., Minggu L. J., Kassim M. B., Amin N. A. S., Salleh W. N. W., Salehmin M. N. I., Nasir M. F. M., Hir Z. A. M. (2018). Appl. Catal., B.

[cit41] Liu J., Zhang T., Wang Z., Dawson G., Chen W. (2011). J. Mater. Chem..

[cit42] Zhang G., Zhang M., Ye X., Qiu X., Lin S., Wang X. (2014). Adv. Mater..

[cit43] Tu W., Xu Y., Wang J., Zhang B., Zhou T., Yin S., Wu S., Li C., Huang Y., Zhou Y., Zou Z., Robertson J., Kraft M., Xu R. (2017). ACS Sustainable Chem. Eng..

[cit44] Dong G., Zhao K., Zhang L. (2012). Chem. Commun..

[cit45] Qin J., Zeng H. (2017). Appl. Catal., B.

[cit46] Wang F., Wang Y., Feng Y., Zeng Y., Xie Z., Zhang Q., Su Y., Chen P., Liu Y., Yao K., Lv W., Liu G. (2018). Appl. Catal., B.

[cit47] Zhao Y., Shalom M., Antonietti M. (2017). Appl. Catal., B.

[cit48] Zhang J., Zhang M., Sun R., Wang X. (2012). Angew. Chem., Int. Ed..

[cit49] Liu G., Zhao G., Zhou W., Liu Y., Pang H., Zhang H., Hao D., Meng X., Li P., Kako T., Ye J. (2016). Adv. Funct. Mater..

