# Supplementary material for: Facile in situ reductive synthesis of both nitrogen deficient and protonated g-C3N4 nanosheets for the synergistic enhancement of visible-light H2 evolution
Source: Chem Sci. 2020 Feb 3;11(10):2716–28. doi: 10.1039/c9sc05060d (PMC8157536; doi:10.1039/c9sc05060d)
Supplement: SC-011-C9SC05060D-s001 [file SC-011-C9SC05060D-s001.pdf]

Electronic Supplementary Material (ESI) for Chemical Science.
This journal is © The Royal Society of Chemistry 2020

Electronic Supplementary Information for

Facile In-situ Reductive Synthesis of Both Nitrogen Deficient and Protonated g-C_3N_4 Nanosheets for the Synergistic Enhancement of Visible-light H_2 Evolution

Weisong Li[a, b], Zheng Guo[a], Litong Jiang[b,c], Lei Zhong[a], Guoning Li[a], Jiajun Zhang[a], Kai Fan[a], Sergio Gonzalez-Cortes[b], Kuijuan Jin[c], Chunjian Xu*[a], Tiancun Xiao*[b] and Peter P. Edwards*[b]

[a]School of Chemical Engineering & Technology, State Key Laboratory of Chemical Engineering (Tianjin University), No. 135, Yaguan Road, Tianjin University, Jinnan District, Tianjin, 300350, China
[b]Inorganic Chemistry Laboratory, University of Oxford, South Parks Road, Oxford, OX1 3QR, United Kingdom
[c]Institute of Physics, Chinese Academy of Sciences, Beijing 100190, China
Correspondence to: Chunjian Xu, Tiancun Xiao, Peter P. Edwards
Email: peter.edwards@chem.ox.ac.uk, xiao.tiancun@chem.ox.ac.uk, cjxu@tju.edu.cn

1. Reagents

Analytical grade reagents including dicyandiamide, NH_4Cl, 85 wt.% H_3PO_4 solution, 50 wt.% H_3PO_2 solution, triethanolamine (TEOA) and chloroplatinic acid hexahydrate (>99%, $H_2PtCl_6{\cdot}6H_2O$) were purchased from Shanghai Macklin biochemical company. K_2HPO_4 (>99%) was obtained from Tianjin Kermel and all these reagents were used without further purification. Deionized water was used in the whole experiment process.

2. Sample characterization

The morphologies of the g-C_3N_4 samples were characterized by the Hitachi S4800 field emission scanning electron microscope (SEM) operating at 3.0 kV and JEOL-2100F transmission electron microscope (TEM). Energy dispersive X-ray analysis (EDX) of the representative samples were also conducted on a scanning transmission electron microscope (STEM, JEM-2100F, JEOL, Japan). N_2 adsorption-desorption isotherms and pore size distributions were obtained at -196 °C with an ASAP2020 Plus HD88 apparatus (Micromeritics, USA), and then the specific areas were calculated using the Brunauer-Emmett-Teller (BET) method. The X-ray diffraction patterns of the samples were collected using a Bruker D8 Focus Diffractometer with Cu Kα as the radiation source. Fourier transform infrared spectroscopy (FTIR) was obtained by a Bruker Tensor-II spectrometer over a range of 4000-450 cm^{-1} with a resolution of 1 cm^{-1}. X-ray photoelectron spectroscopy (XPS) was performed on an ESCALAB 250XI (Thermal Scientific, USA) X-ray photoelectron spectrometer. Electron paramagnetic resonance (EPR) experiments were conducted on a Bruker A300 spectrometer at room temperature. The organic elemental analysis (OEA) of C, N, O and H was performed on Elementar Vario MACRO cube. ^{13}C, ^{31}P and ^{1}H solid-state nuclear magnetic resonance (NMR) spectra were recorded by the Bruker AVIII 600 NMR spectrometer. For the zeta potential measurements, 10 mg of each sample was homogeneously dispersed in 200 mL deionized water using strong ultrasonication and then the zeta potentials were measured at 25 °C with a zeta potential analyzer (Malvern zetasizernano, UK). Shimadzu UV2600 UV-vis spectrometer was used to record the UV-vis absorption spectrums of the samples with $BaSO_4$ as reference. The photoluminescence data excited with 325 nm light source were collected on an Edinburgh Instruments FLS 920 luminescence spectrometer. Time-resolved photoluminescence decay spectra were collected on Edinburgh Instruments FLS 1000 spectrometer with an excitation wavelength of 340 nm.

3. Transient photocurrent measurement

To obtain the photocurrent responses of the samples, working electrodes with g-C_3N_4 coated on the

transparent FTO conductive glass were prepared. Typically, 20 mg sample was dispersed in 20 mL of 50mg/mL naphthalenol/ethanol solution with strong ultrasonication. The dispersed g-C_3N_4 solution was evenly dropped on the conductive side of the FTO glass with a 1×1 cm area and then the electrode was dried in 80 °C oven. The as prepared working electrodes was tested in 1 mol/L Na_2SO_4 aqueous solution and excited by the visible-light (λ≥400nm) irradiation pulse with 20 seconds width, the photocurrent response was recorded by the CHI 660E electrochemical workstation.

4. Photocatalytic H_2 evolution and AQY measurement

The photocatalytic H_2 evolution ability of each sample was evaluated with the RTK-Solar non-vacuum photocatalytic H_2 evolution testing system (RTKINS Ltd. Wuhan, China, **Fig. S1**). This novel H_2 evolution testing system works under atmosphere and the generated H_2 volume can be directly measured by the standard microflow cell with a calibrated volume of 33.7μL, it means that the volume of each bubble flowing through the microflow cell is 33.7 μL. Then, the number of bubbles flowing through the microflow cell will be counted by a photoelectric counter and recorded on the workstation installed on a computer, subsequently obtaining the volume of the generated H_2. Thus, with the system temperature being maintained constant using a waterbath, the amount of the generated H_2 can be calculated by the ideal gas equation of state PV=*n*RT. Fig. S1 shows the composing parts this testing system. The whole reactor is surrounded by cooling water, thereby ensuring the system temperature maintained constant.

The photocatalytic H_2 generation rate of each sample was measured with 1.5 wt% of Pt loading. The detailed operating procedures were as following: 1) 15mg sample was homogeneously dispersed in 80mL of 20 vol.% TEOA solution using strong ultrasonication; 2) 1.2 g of 0.5 mg/mL H_2PtCl_6 solution (stabilized with 5 wt.% HCl) was added to the above catalyst solution and then transferred to the specially designed H_2 evolution reactor (reactor volume=120 mL; 3) the reactor was tightly sealed and purged using high purity N_2 for 30mins to remove the oxygen in the solution and system; 4) Stop N_2 purging and ensure that there was no gas leakage in the system; 5) Start visible-light irradiation (λ≥400nm) using the 300 W Xe lamp (Aulight, China) with a 400 nm cutoff filter and the reactor temperature was kept at 20 ℃ using a constant temperature waterbath. 6) After irradiation for 1 hour to insure that the Pt photoreduction loading process was completed, start the RTK-Solar workstation to record the volume of generated H_2. For the H_2 evolution experiments conducted with K_2HPO_4 addition, the K_2HPO_4 solid was premixed with 20 vol.% TEOA solution. For all experiments, the reactor was wrapped with foil to reduce light leakage (as shown in the inset of Fig. S1). Then, the molar amount of the generated H_2 ($V_{\rm mol}$) can be calculated according to Equation S1 and S2:

$$V_{mol} = \frac{\mathrm{RT}}{\mathrm{P}} = \frac{8.314 \times 293.15}{101.325} = 24.05\ \mathrm{mL/mmol} \qquad \text{(Equation S1)}$$

$$n = \frac{\mathrm{V}}{V_{mol}} = \frac{\mathrm{V}}{24.05} \qquad \text{(Equation S2)}$$

Here, V represents the volume of the generated H_2, mL; *n* represents the H_2 molar amount, mmol.

The AQY of the 0.8-P2-CN sample at 420 nm was measured with the Xe lamp equipped an 420±10 nm monochromatic filter, and all the other details were the same with those in the H_2 evolution experiments. The light intensity was measured with the same procedures in our previous work[1] and the calculated light intensity was 2.93 mW/cm^2. The calculated light irradiation area was 33.18 cm^2. The H_2 evolution rates over 1.5 wt.% Pt-loaded 0.8-P2-CN in 20 vol.% TEOA and 20 vol.% TEOA+32 mmol K_2HPO_4 solution were 65.9 and 248.1 μmol·h^{-1}, respectively **(**Fig. S6**)**. Thus, the AQY calculated according to Equation S3 for 0.8-P2-CN catalyzed H_2 evolution in 20 vol.% TEOA and 20 vol.% TEOA+32 mmol K_2HPO_4 solutions were 10.7 % and 40.4 %, respectively.

$$\text{AQY}(\%) = \frac{\text{the number of reacted electrons}}{\text{the number of incident photons}} \times 100$$
$$= \frac{\text{the number of evolved } H_2 \text{ molecules} \times 2}{\text{the number of incident photons}} \times 100$$
$$= \frac{\text{evoluted } H_2 \text{ per hour } (\mu\text{mol}) \times 6.02 \times 10^{23} \times 10^{-6} \times 2}{\frac{2.93 \times 10^{-3} \times 33.18 \times 3600}{\frac{1240}{420} \times 1.6 \times 10^{-19}}} \times 100$$

(Equation S3)

5. Energy changes (Δ*E*) calculation for removing a lattice N atom in the g-C_3N_4 basic melon framework with PH_3

The energy changes (Δ*E*) for removing a lattice N atom from N1, N2, N3 and N4 were calculated as Equation S4 and S5. The reduction reactions using H_2 and PH_3 as reduction agents could be illustrated as Equation S6 and S7.

$$\Delta \boldsymbol{E} = \boldsymbol{E}[\text{Reduced melon}] + \boldsymbol{E}[NH_3] - \boldsymbol{E}[\text{melon}] - \boldsymbol{E}[PH_3]$$ (Equation. S4)

$$\Delta \boldsymbol{E} = \boldsymbol{E}[\text{Reduced melon}] + \boldsymbol{E}[NH_3] - \boldsymbol{E}[\text{melon}] - \boldsymbol{E}[H_2] + \boldsymbol{E}[H_2] - \boldsymbol{E}[PH_3]$$ (Equation. S5)

$$\textbf{C-NH}_{\boldsymbol{x}} + (6-2\boldsymbol{x})/3\ \textbf{H}_2 \rightarrow \textbf{NH}_3 + \textbf{CH}_{(3-4x)/3}\ (\boldsymbol{x}=0, 1 \text{ or } 2)$$ (Equation. S6)

$$\textbf{C-NH}_{\boldsymbol{x}} + (6-2\boldsymbol{x})/3\ \textbf{PH}_3 \rightarrow \textbf{NH}_3 + \textbf{CH}_{(3-2x)} + (6-2\boldsymbol{x})/3\ \textbf{P}\ (\boldsymbol{x}=0, 1 \text{ or } 2)$$ (Equation. S7)

The energy changes for removal of one N atom at N1, N2, N3 and N4 positions and terminating the dangling carbon atoms in H_2 atmosphere were taken from literature**[2]** and as follows:

$$\Delta \boldsymbol{E}\ (\text{N1}, H_2) = 0.65\ \text{eV}$$

$$\Delta \boldsymbol{E}\ (\text{N2}, H_2) = 0.83\ \text{eV}$$

$$\Delta \boldsymbol{E}\ (\text{N3}, H_2) = 1.40\ \text{eV}$$

$$\Delta \boldsymbol{E}\ (\text{N4}, H_2) = 2.39\ \text{eV}$$

According to literature**[3]**, ***E***[H_2]=0 eV, ***E***[PH_3]=5.6 kJ/mol=0.06 eV (white phosphorus as standard, ***E***[P]=0 eV). Thus, the energy changes for removing one N atom at the four positions using PH_3 were calculated as following:

$$\Delta \boldsymbol{E}\ (\text{N1}, PH_3) = \Delta \boldsymbol{E}\ (\text{N1}, H_2) + \boldsymbol{E}[H_2] - 2/3\boldsymbol{E}[PH_3] = 0.65 - 0.06 \times 2/3 = 0.61\ \text{eV}$$

$$\Delta \boldsymbol{E}\ (\text{N2}, PH_3) = \Delta \boldsymbol{E}\ (\text{N2}, H_2) + 2\boldsymbol{E}[H_2] - 4/3\boldsymbol{E}[PH_3] = 0.83 - 0.06 \times 4/3 = 0.75\ \text{eV}$$

$$\Delta \boldsymbol{E}\ (\text{N3}, PH_3) = \Delta \boldsymbol{E}\ (\text{N3}, H_2) + 3\boldsymbol{E}[H_2] - 2\boldsymbol{E}[PH_3] = 1.40 - 0.06 \times 2 = 1.28\ \text{eV}$$

$$\Delta \boldsymbol{E}\ (\text{N4}, PH_3) = \Delta \boldsymbol{E}\ (\text{N4}, H_2) + 3\boldsymbol{E}[H_2] - 2\boldsymbol{E}[PH_3] = 2.39 - 0.06 \times 2 = 2.27\ \text{eV}$$

6. Accuracy verification of the RTK-Solar H_2 evolution system

The verification contained two steps. Firstly, two H_2 evolution experiments (using 1.5 wt.% Pt loaded 0.8-P2-CN as catalysts) in the 20 vol.% TEOA and 20 vol.% TEOA+32 mmol K_2HPO_4 mixture solutions, respectively. The operating procedures were the similar with those in the previous manuscript. In each experiment, the reaction system was purged by high purity N_2 (99.99%) and 4 mL helium was added as internal standard. The reactor was tightly sealed and irradiated under visible light (λ≧400 nm) for 120 mins. After reaction, the reactor was connected to a Hiden HPR 20 gas chromatograph/mass spectrometer system and the gases were carefully checked. As shown in Fig. S6, the mass spectrums

indicated that only H_2 was detected excluding the purging gas N_2 and internal standard Helium. Thus, the volume measured in the RTK-Solar system can be directly regarded as the volume of photocatalytic evolved H_2.

Secondly, to verify the accuracy of the H_2 evolution results obtained by the RTK-Solar system, parallel experiments were conducted with the reactor being connected to a closed gas-circulation system and the generated H_2 was determined by a calibrated gas chromatography equipped TCD detector (Clarus 580, PerkinElmer, Helium as carrier gas). All the experimental conditions were the same with those in the RTK-Solar system. The system was purged by high purity N_2 (99.99%) and 5 mL Argon was injected into the system as internal standard. The 1.5 wt.% Pt loaded B-CN, G-CN, 1.6-P1-CN and 0.8-P2-CN were tested in the 20 vol.% TEOA solution and the gas sample was analyzed for every 1 hour. Table S2 and Fig. S7 presented the summarized gas chromatography original data and curves for the H_2 evolution tests, respectively. The full original gas chromatography curves can be found with the following link:

https://www.dropbox.com/s/4k5j46w5rdkuejp/GC%20original%20data%20of%20the%20supplemented%20H2%20evolution%20tests.pdf?dl=0

The generated H_2 amount determined by the gas chromatography method can be calculated by the following equation:

$$n_{H_2} = \frac{H_2(\text{adjusted}) \times 5 \times 1000}{\text{Argon}(\text{adjusted}) \times 22.4} \quad \mu\text{mol}$$

According to the summarized gas chromatograph data, the evolved H_2 amount can be calculated. Then, H_2 evolution vs time plot can be also illustrated in **Fig. S7**. Based on the linear fitting results, the gas chromatography determined H_2 evolution rates over the 1.5 wt.% Pt loaded B-CN, G-CN, 1.6-P1-CN and 0.8-P2-CN in the 20 vol.% TEOA solution were 42.8, 106.6, 140.1 and 261.6 $\mu mol \cdot h^{-1}$, respectively. Thus, the ratios of the H_2 evolution rates determined by gas chromatography and RTK-Solar system were located in a narrow range of 0.972~1.076. It means that the RTK-Solar measured H_2 evolution rate is very close to that determined by the widely accepted gas chromatography method. In the other word, this novel RTK-Solar H_2 evolution system has adequate accuracy to measure the amount of the generated gases. Combining with the mass spectrums and gas chromatography data, the H_2 evolution rates determined using the novel RTK-Solar H_2 evolution system should be reliable. In addition, the Effect of photocatalyst amount on the H_2 evolution was explored and the results were presented in **Fig. S9** and the results indicated that it was suitable to fix the photocatalyst usage at 15 mg for each experiment.

7. Supporting figures

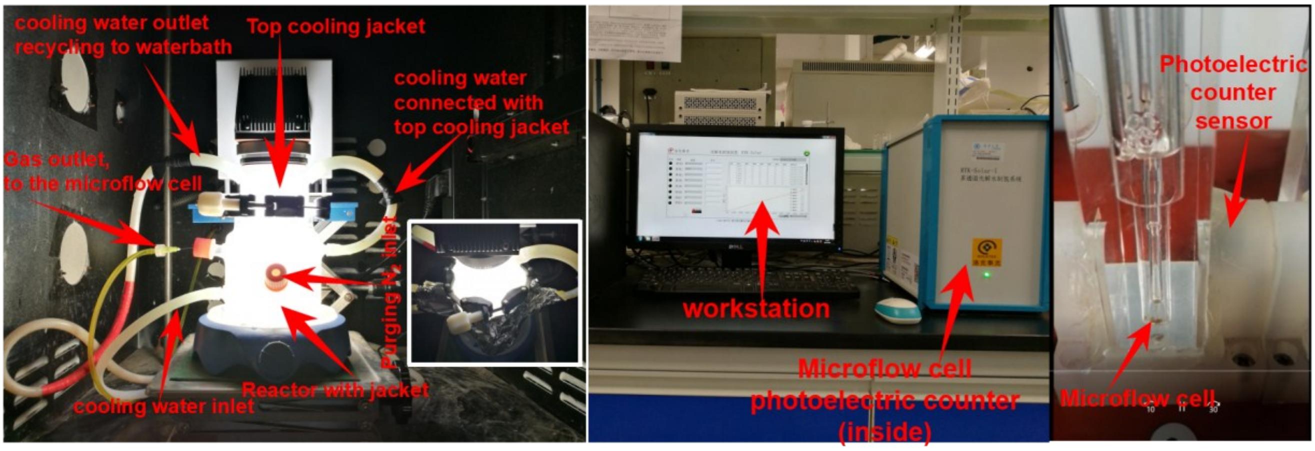

Fig. S1. Digital pictures of the photocatalytic H_2 evolution testing system

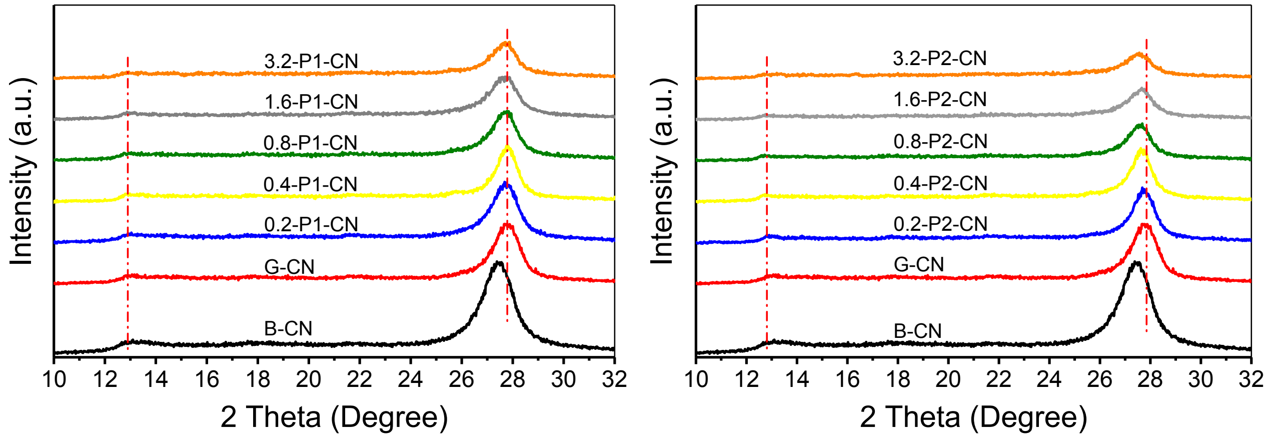

Fig. S2. Further comparisons between the as prepared g-C_3N_4 samples

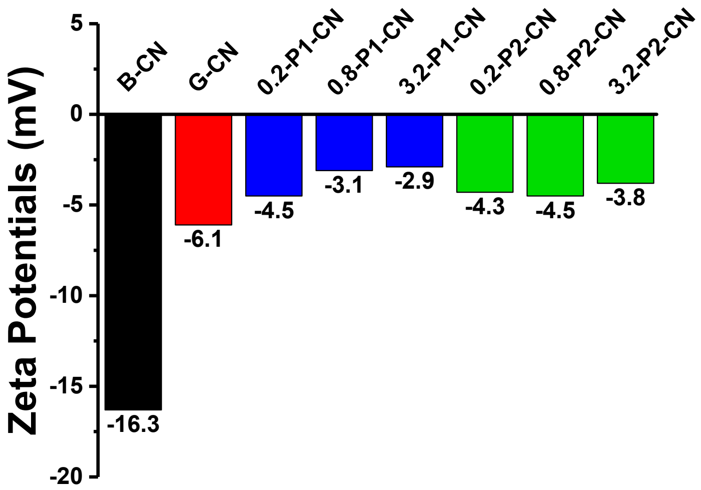

Fig. S3. Measured Zeta potentials of the g-C_3N_4 samples

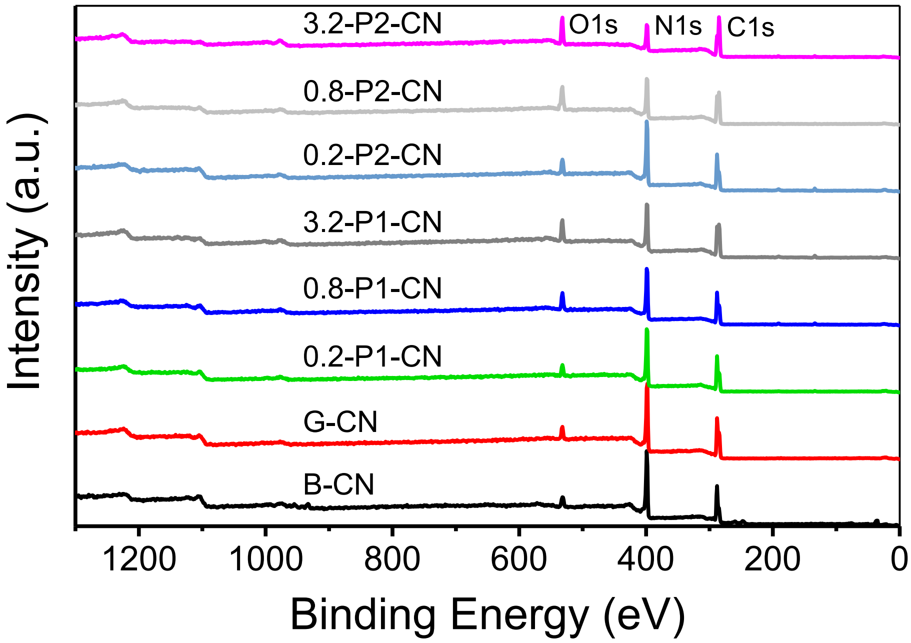

Fig. S4. XPS spectra of the g-C_3N_4 samples

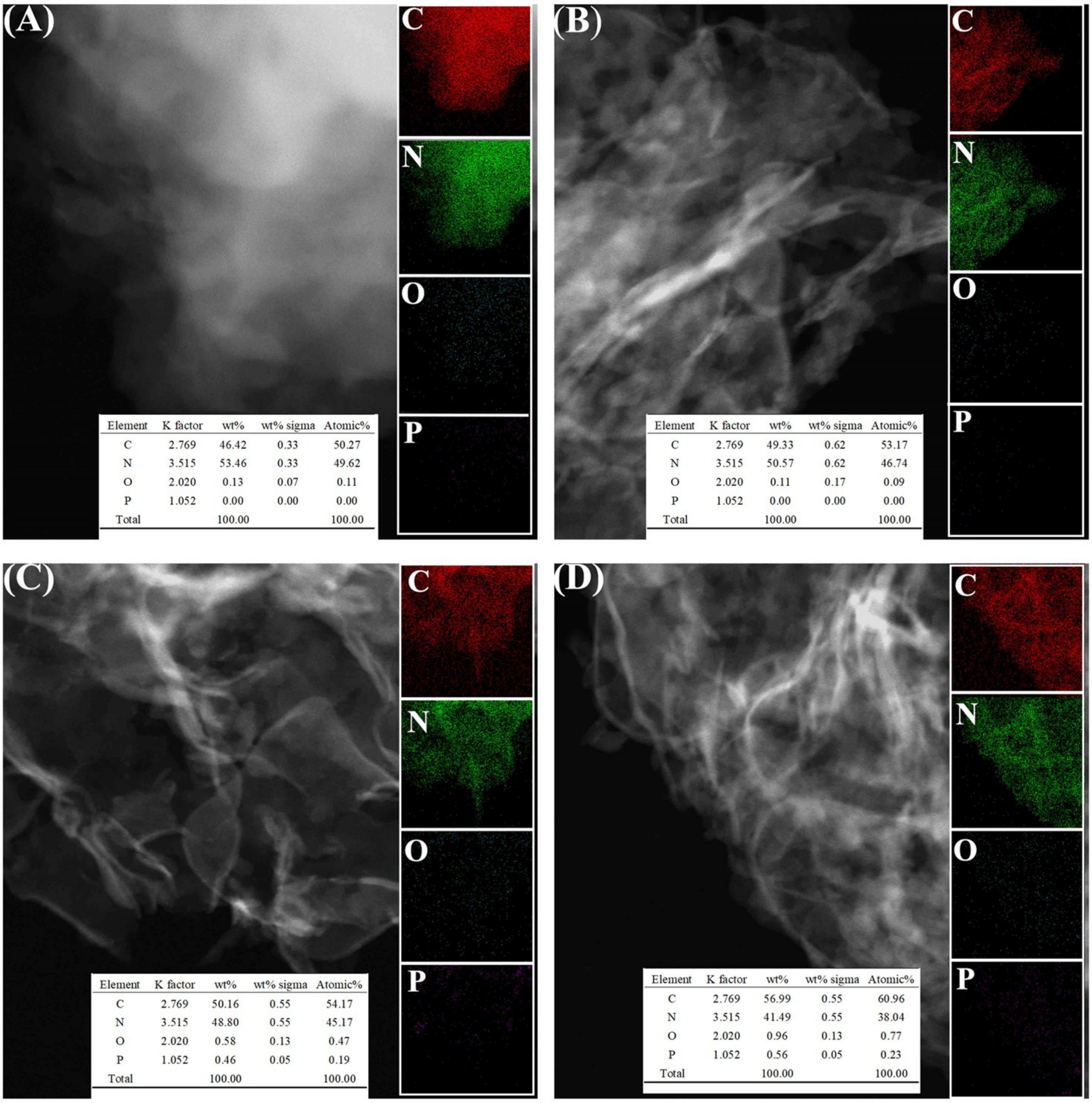

(A)

Element	K factor	wt%	wt% sigma	Atomic%
C	2.769	46.42	0.33	50.27
N	3.515	53.46	0.33	49.62
O	2.020	0.13	0.07	0.11
P	1.052	0.00	0.00	0.00
Total		100.00		100.00

(B)

Element	K factor	wt%	wt% sigma	Atomic%
C	2.769	49.33	0.62	53.17
N	3.515	50.57	0.62	46.74
O	2.020	0.11	0.17	0.09
P	1.052	0.00	0.00	0.00
Total		100.00		100.00

(C)

Element	K factor	wt%	wt% sigma	Atomic%
C	2.769	50.16	0.55	54.17
N	3.515	48.80	0.55	45.17
O	2.020	0.58	0.13	0.47
P	1.052	0.46	0.05	0.19
Total		100.00		100.00

(D)

Element	K factor	wt%	wt% sigma	Atomic%
C	2.769	56.99	0.55	60.96
N	3.515	41.49	0.55	38.04
O	2.020	0.96	0.13	0.77
P	1.052	0.56	0.05	0.23
Total		100.00		100.00

Fig. S5. Elemental mapping collected using scanning transmission electron microscopy. A) B-CN; B) G-CN; C) 0.8-P1-CN and D) 0.8-P2-CN, respectively.

8. Density-Functional-Theory (DFT) calculations

Electronic band structure of B-CN and other g-C_3N_4 samples were carried out using general-gradient-approximation-Perdew-Burke-Ernzerhof (PBEsol) and projector augmented-wave (PAW) method[4] as implemented in the Vienna Ab initio simulation package (VASP)[5]. The energy cutoff is 500 eV. Here 3×4×2-centered k-point grids were used to integrate the Brillouin zone. A two dimensional (2D) melon sheet was constructed to represent the incomplete polycondensation of melon, with a large vacuum space of 24 Å to separate two neighboring 2D melon sheets. A cubic cell was adopted for the carbon nitrides materials with experimental lattice constants of a = 16.7 Å and b = 12.4 Å[6]. The representative cell units are presented as in Fig. S6.

For better understanding, basic atoms composing the g-C_3N_4 "melon" structure (Fig. S6A), the proposed groups and vacancies were clearly marked. As seen in Fig. S6B, the carboxyl groups which originated from the N1 or N2 nitrogen atoms (referring to Fig. 3A in the manuscript) removal and heptazine ring opening, are located at the apex of g-C_3N_4 "melon" structure. While, Fig. S6C presents the other possible nitrogen vacancy which is originated from N3 nitrogen atoms loss.

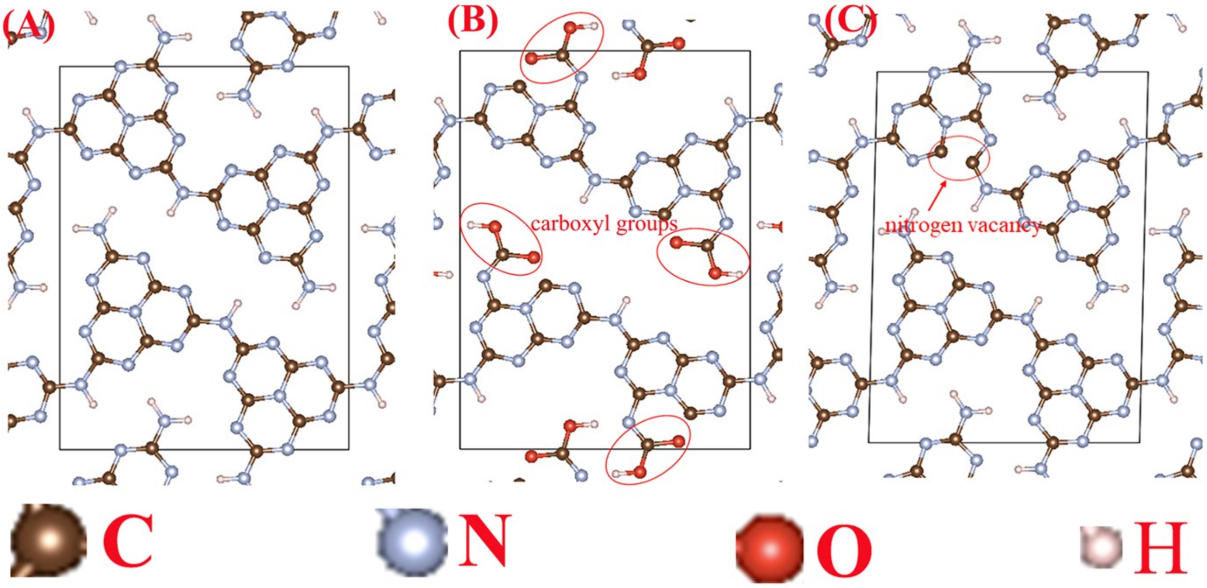

Fig. S6. A) Cubic cell structure of B-CN. B)g-C_3N_4 cell only containing carboxyl groups. C) g-C_3N_4 cell only containing nitrogen vacancies.

9. Bandgap structures

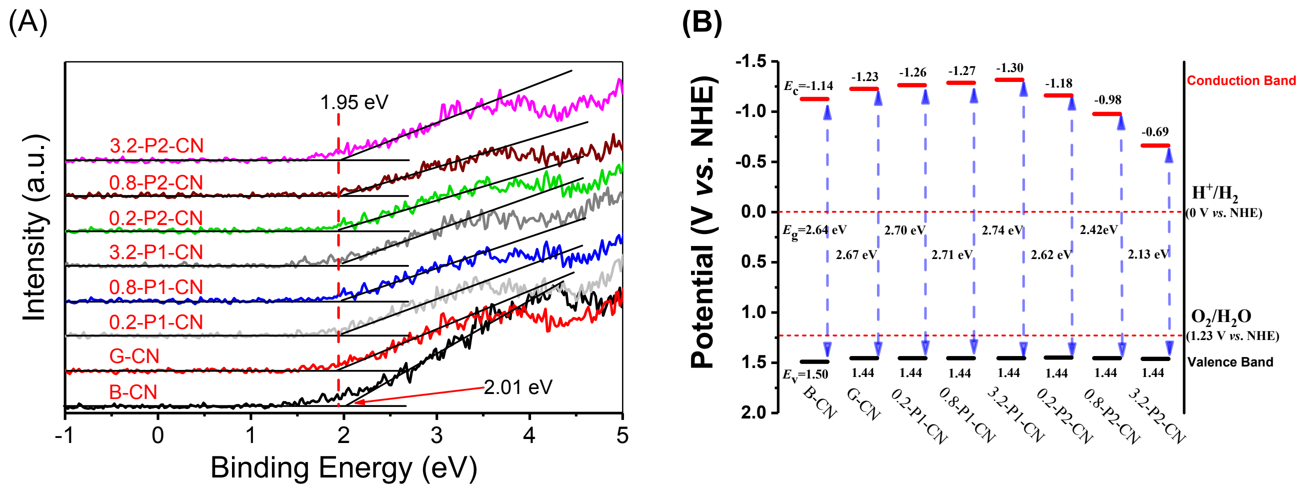

Fig. S7. A) XPS valence band spectra. B) Band structures alignments of the g-C_3N_4 samples. The contact differences between samples and analyzer versus normal hydrogen electrode (NHE) at PH=7 were determined with the formula of E_{NHE}/V=Φ+VB-4.44 (Φ=3.93 eV, representing the electron work function of the analyzer).

10. RTK H_2 evolution reactor accuracy verification results

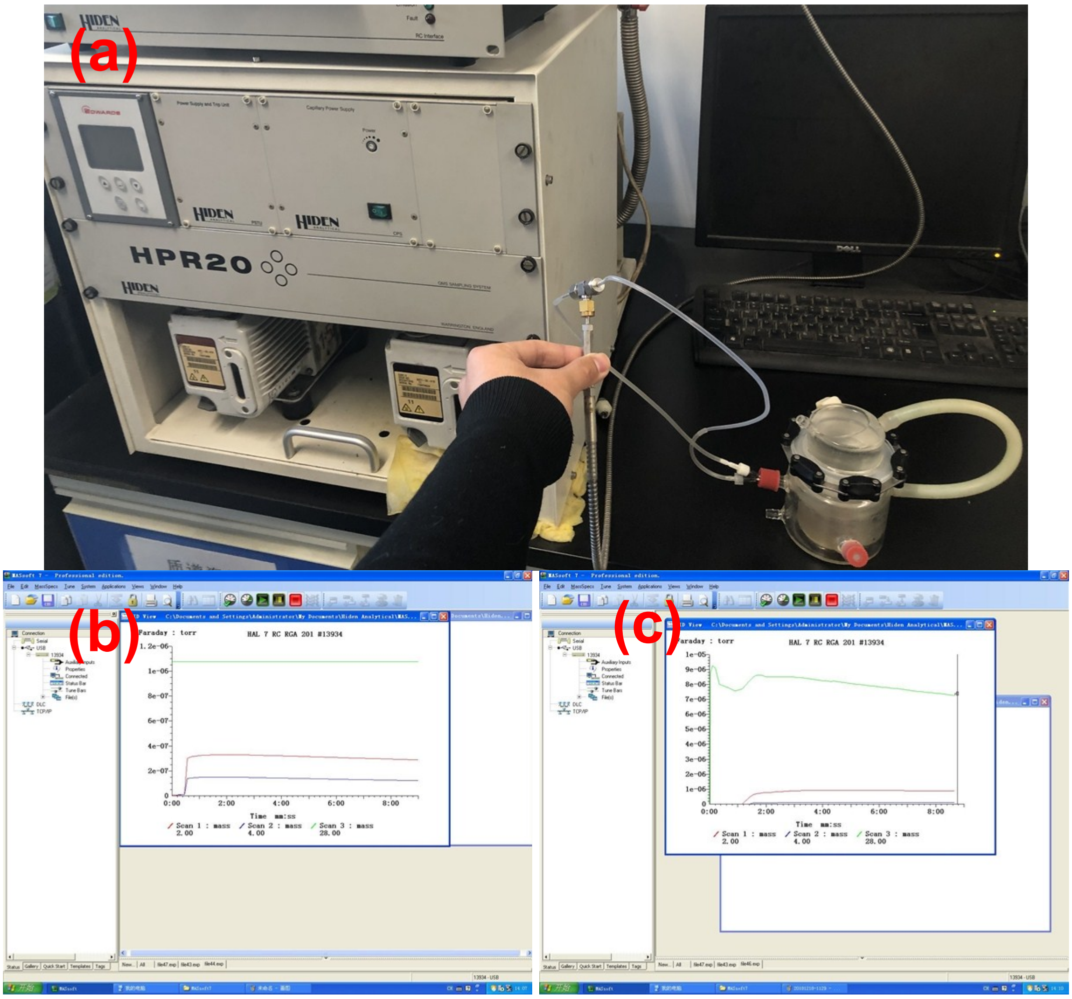

Fig. S8. Hiden HPR 20 gas chormatograph/mass spectrometer system for the Pt loaded g-C_3N_4 photocatalytic H_2 evolution system: (a) Equipment picture; (b) and (c) were the mass spectrums of the product gases in 20 vol.% TEOA and 20 vol.% TEOA+32 mmol K_2HPO_4 mixture solution, respectively.

(a)

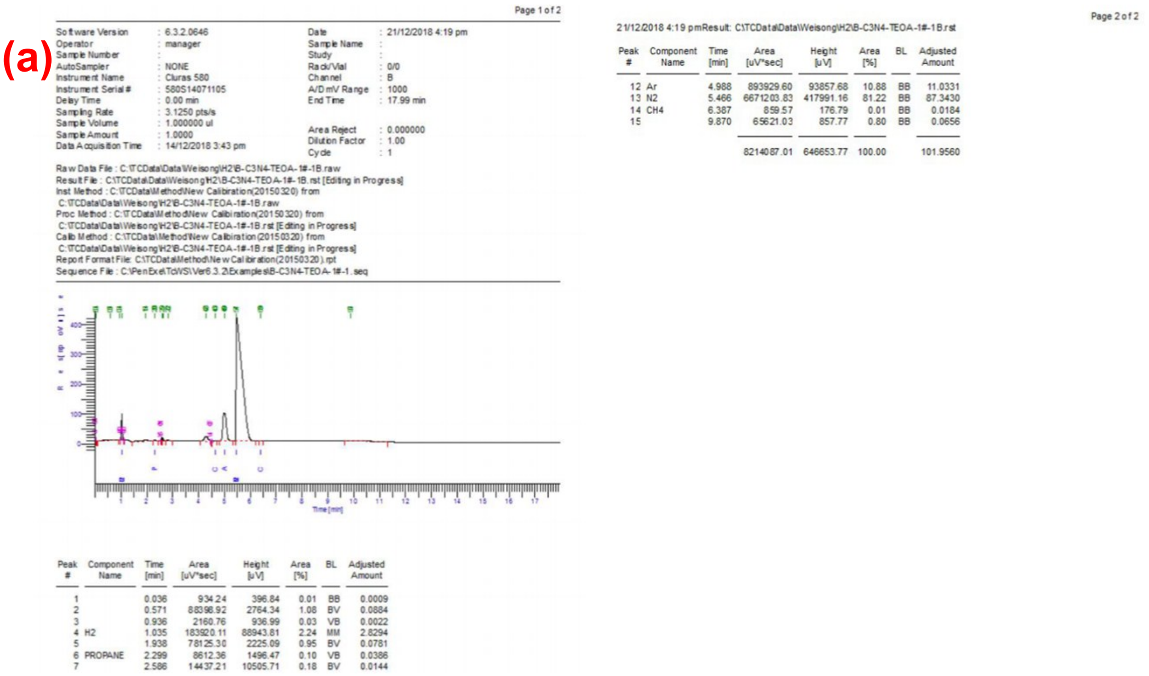

Page 1 of 2

Software Version	: 6.3.2.0646	Date	: 21/12/2018 4:19 pm
Operator	: manager	Sample Name	:
Sample Number	:	Study	:
AutoSampler	: NONE	Rack/Vial	: 0/0
Instrument Name	: Cluras 580	Channel	: B
Instrument Serial #	: 580S14071105	A/D mV Range	: 1000
Delay Time	: 0.00 min	End Time	: 17.99 min
Sampling Rate	: 3.1250 pts/s		
Sample Volume	: 1.000000 ul	Area Reject	: 0.000000
Sample Amount	: 1.0000	Dilution Factor	: 1.00
Data Acquisition Time	: 14/12/2018 3:43 pm	Cycle	: 1

Raw Data File : C:\TCData\Data\Weisong\H2\B-C3N4-TEOA-1#-1B.raw
Result File : C:\TCData\Data\Weisong\H2\B-C3N4-TEOA-1#-1B.rst [Editing in Progress]
Inst Method : C:\TCData\Method\New Calibration(20150320) from
C:\TCData\Data\Weisong\H2\B-C3N4-TEOA-1#-1B.raw
Proc Method : C:\TCData\Method\New Calibiration(20150320) from
C:\TCData\Data\Weisong\H2\B-C3N4-TEOA-1#-1B.rst [Editing in Progress]
Calib Method : C:\TCData\Method\New Calibration(20150320) from
C:\TCData\Data\Weisong\H2\B-C3N4-TEOA-1#-1B.rst [Editing in Progress]
Report Format File: C:\TCData\Method\New Calibiration(20150320).rpt
Sequence File : C:\PenExe\TcWS\Ver6.3.2\Examples\B-C3N4-TEOA-1#-1.seq

Peak #	Component Name	Time [min]	Area [uV*sec]	Height [uV]	Area [%]	BL	Adjusted Amount
1		0.036	934.24	396.84	0.01	BB	0.0009
2		0.571	88398.92	2764.34	1.08	BV	0.0884
3		0.936	2160.76	936.99	0.03	VB	0.0022
4	H2	1.035	183920.11	88943.81	2.24	MM	2.8294
5		1.938	78125.30	2225.09	0.95	BV	0.0781
6	PROPANE	2.299	8612.36	1496.47	0.10	VB	0.0386
7		2.586	14437.21	10505.71	0.18	BV	0.0144

Page 2 of 2

21/12/2018 4:19 pmResult: C:\TCData\Data\Weisong\H2\B-C3N4-TEOA-1#-1B.rst

Peak #	Component Name	Time [min]	Area [uV*sec]	Height [uV]	Area [%]	BL	Adjusted Amount
12	Ar	4.988	893929.60	93857.68	10.88	BB	11.0331
13	N2	5.466	6671203.83	417991.16	81.22	BB	87.3430
14	CH4	6.387	859.57	176.79	0.01	BB	0.0184
15		9.870	65621.03	857.77	0.80	BB	0.0656
			8214087.01	646653.77	100.00		101.9560

(b)

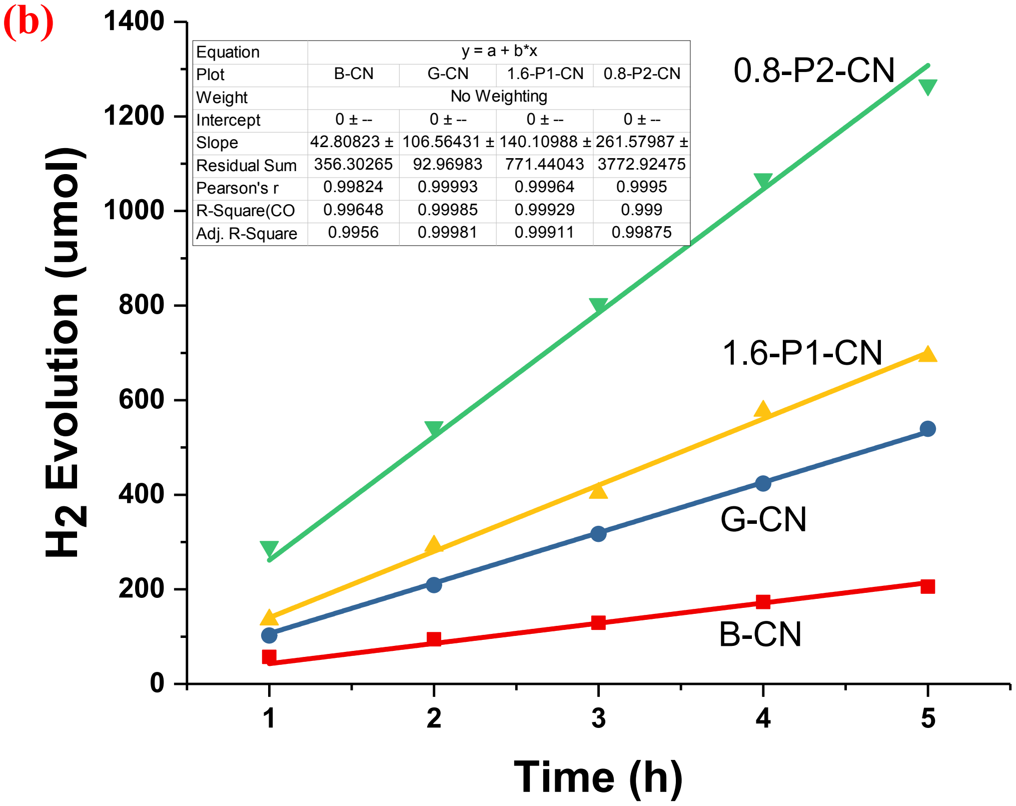

Fig. S9. (a) Original gas chromatography curve for the H_2 evolution experiments; (b) H_2 evolution vs time plot over the Pt loaded representative g-C_3N_4 samples in the 20 vol.% TEOA solution

11. Surface properties comparisons of the fresh and used 0.8-P2-CN nanosheets

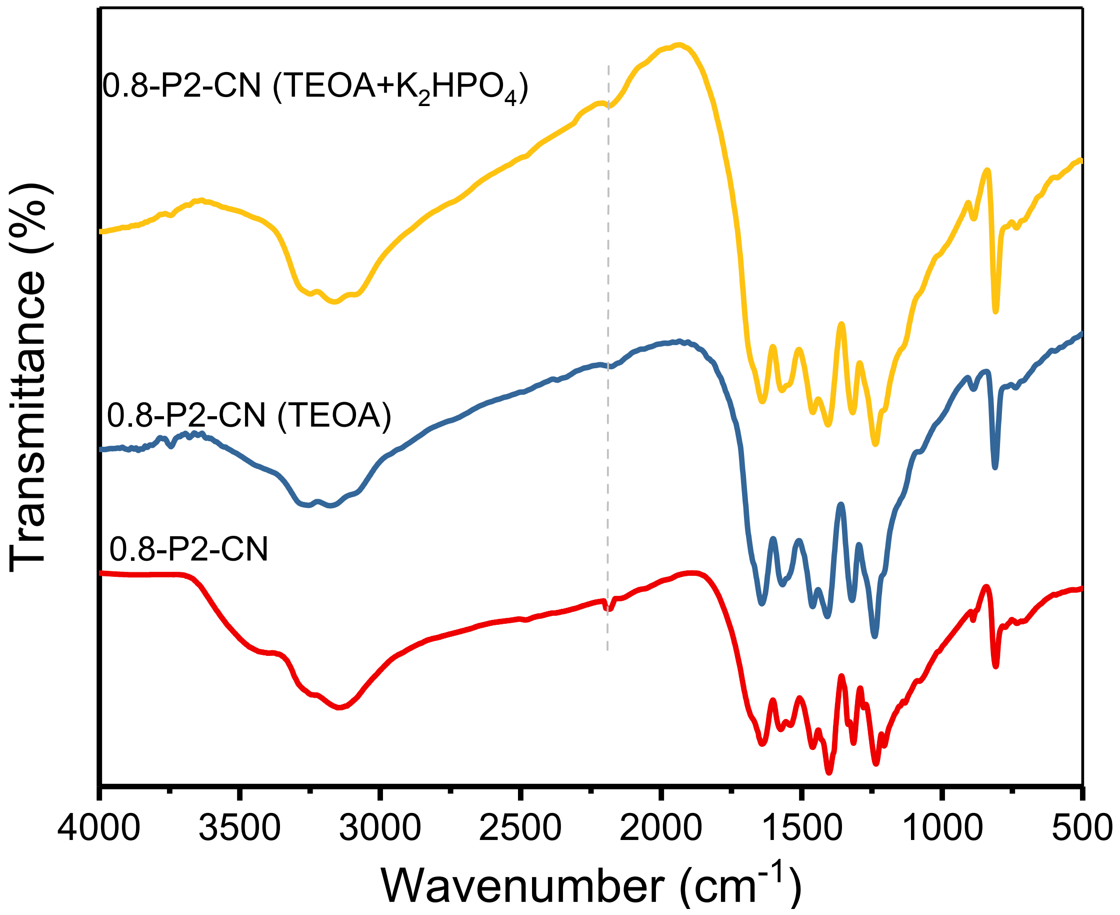

Fig. S10. FTIR spectra of the fresh and used 0.8-P2-CN nanosheets

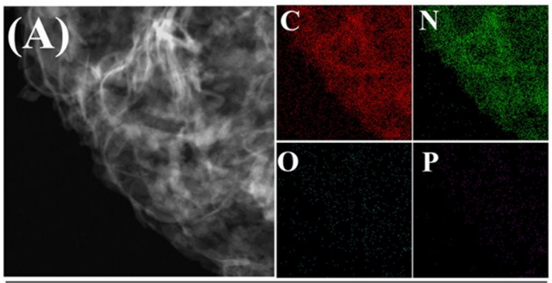

Element	K factor	wt%	wt% sigma	Atomic%
C	2.769	56.99	0.55	60.96
N	3.515	41.49	0.55	38.04
O	2.020	0.96	0.13	0.77
P	1.052	0.56	0.05	0.23
Total		100.00		100.00

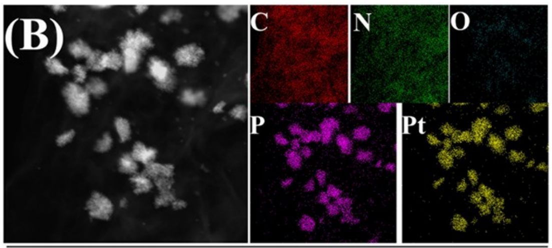

Element	K factor	wt%	wt% sigma	Atomic%
C	2.769	47.16	0.45	66.64
N	3.515	25.05	0.43	30.35
O	2.020	0.48	0.11	0.51
P	1.052	0.28	0.16	0.15
Pt	2.266	27.03	0.39	2.35
Total		100.00		100.00

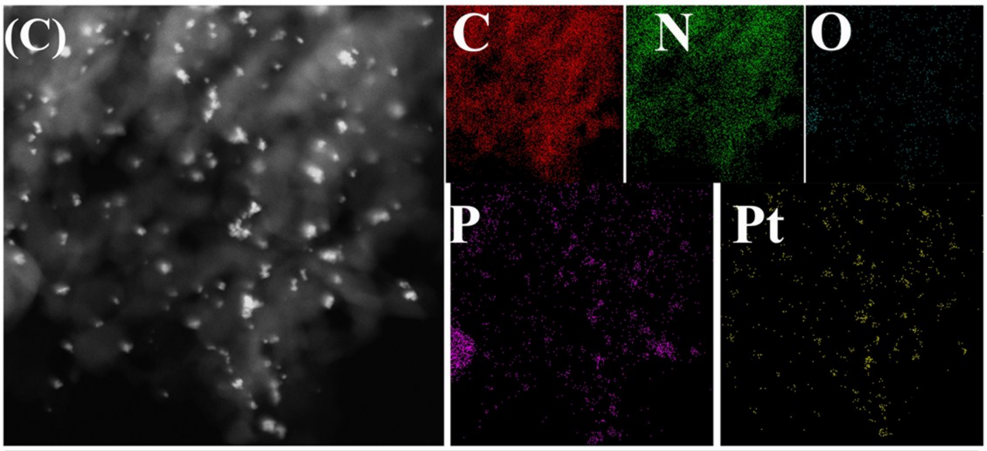

Element	K factor	wt%	wt% sigma	Atomic%
C	2.769	61.38	0.47	66.99
N	3.515	34.07	0.47	31.89
O	2.020	0.52	0.10	0.42
P	1.052	1.20	0.08	0.51
Pt	2.266	2.84	0.17	0.19
Total		100.00		100.00

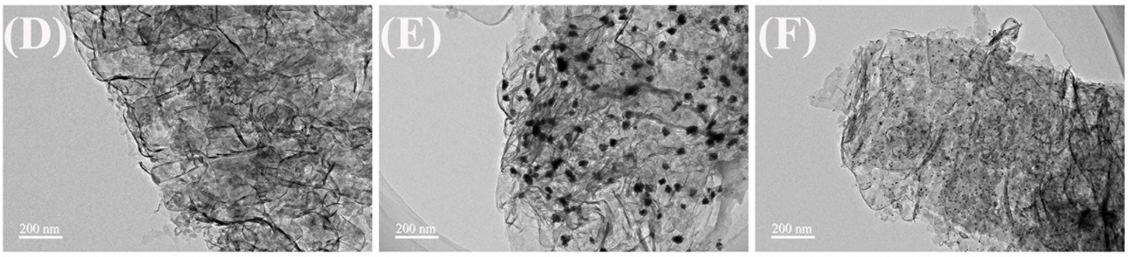

Fig. S11. EDX and TEM analysis of the fresh and used 0.8-P2-CN nanosheets. A) and D) are the elemental mapping and TEM images of fresh 0.8-P2-CN, respectively. B) and E) are the elemental mapping and TEM images of used 0.8-P2-CN after H_2 evolution in 20 vol.% TEOA solution, respectively. C) and F) are the mapping and TEM results of the used 0.8-P2-CN after H_2 evolution experiment in 20 vol.% TEOA/K_2HPO_4 mixed solution

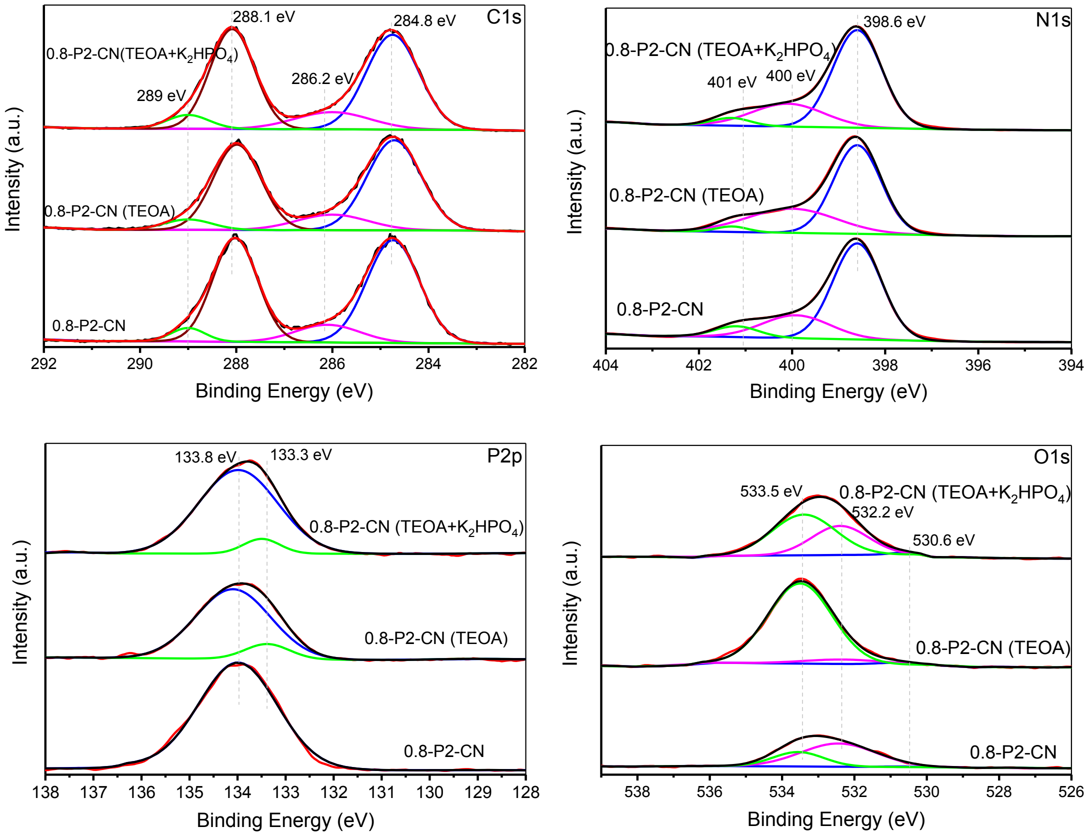

Fig. S12. Narrow XPS scan of the fresh and used 0.8-P2-CN nanosheets. In the figure, (TEOA) and ($TEOA+K_2HPO_4$) represent that the H_2 evolution experiment were conducted in TEOA and TEOA/K_2HPO_4 mixed solution, respectively.

As seen in Fig. S10, the FTIR spectra indicated that the H_3PO_2 doped 0.8-P2-CN nanosheets could well conserve the cyano group signal after photocatalytic H_2 evolution. The elemental mapping shown in Fig. S11 also revealed that the compositions of the g-C_3N_4 could maintain at the same level as before H_2 evolution experiments. The XPS spectra in Fig. S12 indicate that C and N are kept the same after reaction. There is a small 133.8 eV peak shift towards lower binding energy in the P2p spectra of the used 0.8-P2-CN nanosheets, which should be attributed to the interaction of the doped phosphorus with deposited Pt nanoparticles. This coincides with the interesting phenomenon observed in the elemental mapping, in which the distribution of Pt is highly consistent with that of the doped phosphorus (Fig. S11B and S11C).

In brief, the surface properties comparison between the used and fresh 0.8-P2-CN indicate that there are no obvious changes which may induce adverse effect on the performance of the samples. Thus, the stability of 0.8-P2-CN could be further evidenced.

12. AQY results of the H_2 evolution experiments

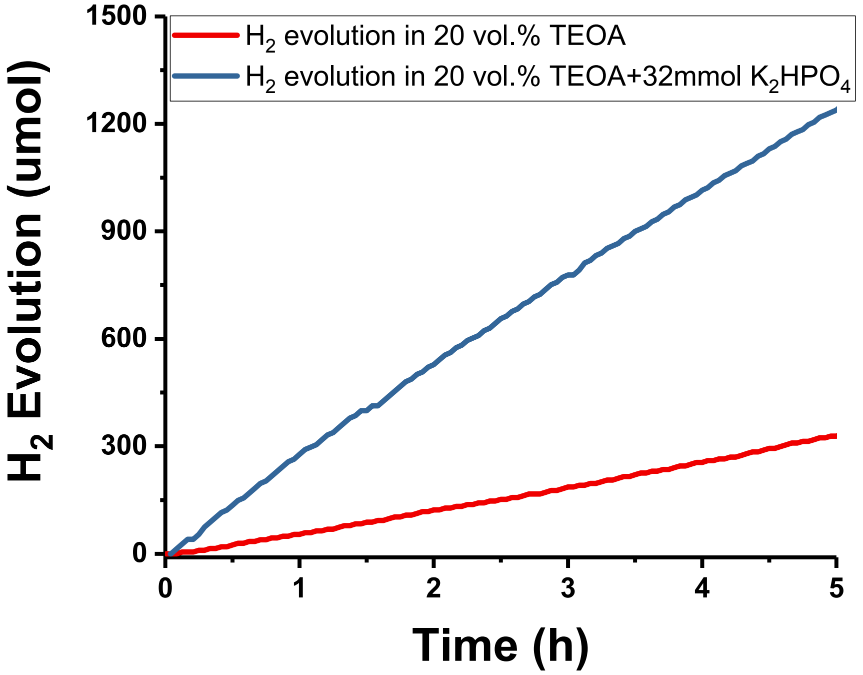

Fig. S13. Time course of H_2 evolution in the experiments for measuring the AQY of 0.8-P2-CN nanosheets under 420±10 nm monochromatic light irradiation

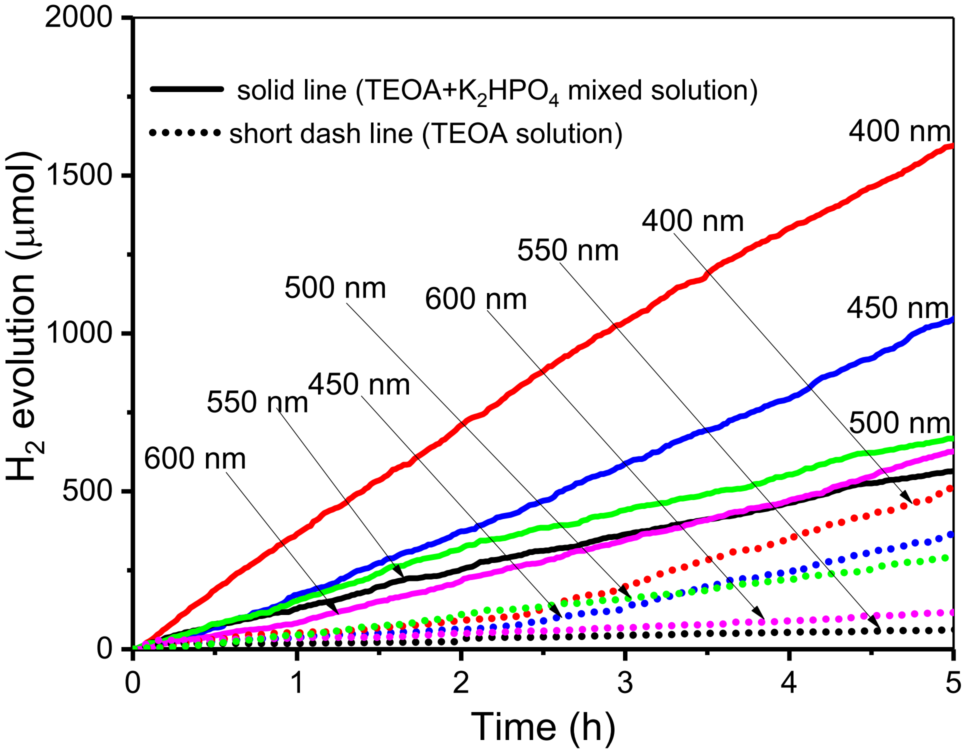

Fig. S14. Time course of H_2 evolution for measuring the AQY of 0.8-P2-CN nanosheets under 400, 450, 500, 550 and 600 nm monochromatic light irradiation. The short dash lines represent the H_2 evolution using 20 vol.% TEOA solution as sacrificing agent, and the solid lines represent the experiments were conducted in optimized TEOA/K_2HPO_4 mixed solution.

13. Other details for the H_2 evolution experiments

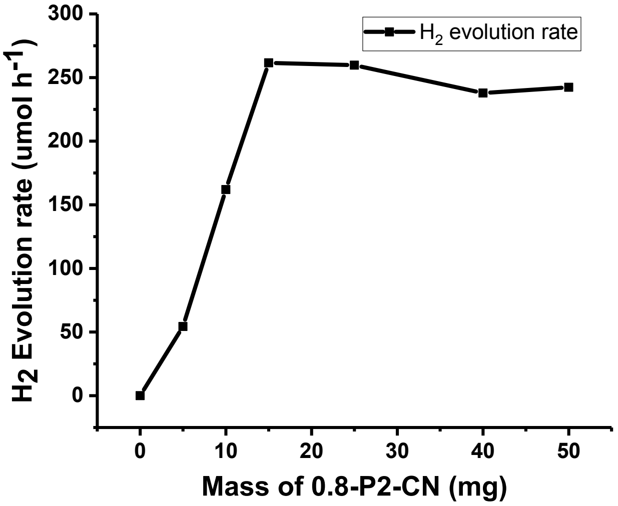

Fig. S15. Effect of photocatalyst amount on the H_2 evolution over 1.5 wt.% Pt loaded 0.8-P2-CN in 20 vol.% TEOA solution under visible-light (λ≧400 nm) irradiation. The H_2 evolution rate increases with the increasing amount of 0.8-P2-CN in the range of 0~15 mg, and the H_2 evolution rate at 15 mg catalyst was very close to that of 25 mg 0.8-P2-CN. While, with further addition of g-C_3N_4, the H_2 evolution rate will slightly go down. It indicates that it is suitable to fix the photocatalyst usage at 15 mg.

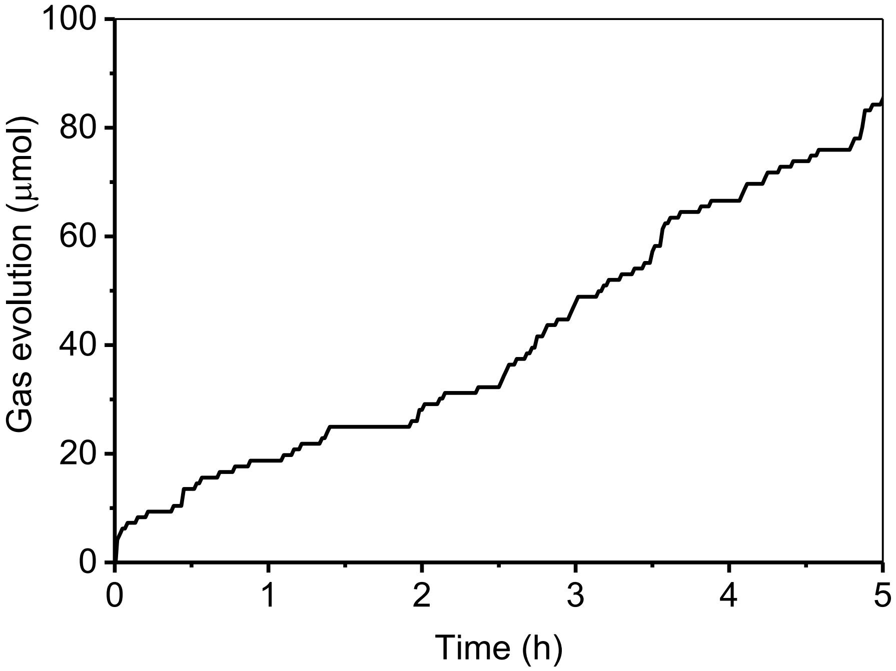

Fig. S16. H_2 evolution control experiment in 20 vol.% H_3PO_4 solution over 0.8-P2-CN nanosheets (Pt was added as cocatalyst)

14. Supporting Tables

Table S1. Summarized XPS data for the as prepared g-C_3N_4 samples. C1s, N1s and O1s binding energies for the selected carbon, nitrogen and oxygen species, respectively, and N/C and O/C ratios determined from the quantitative analysis were provided:

Samples	Binding Energy (eV)								Ratios				
	N-C=N	C-NH_x or C≡N	C-C or C=C	NH_x	N_{3C}	N_{2C}	OH	C-O	NH_x/C	N_{3C}/C	N_{2C}/C	C-O/C	OH/C
B-CN	288.2	286.4	284.8	401	400.1	398.6	533.5	532.2	0.145	0.119	1.054	0.015	0.009
G-CN	288.1	286.5	284.8	401	400	398.6	533.5	532.2	0.135	0.231	0.917	0.039	0.023
0.8-P1-CN	288.1	286.5	284.8	401	400	398.6	533.5	532.2	0.079	0.305	0.882	0.070	0.031
0.8-P2-CN	288.1	286.1	284.8	401	399.3	398.6	533.5	532.2	0.064	0.405	0.568	0.134	0.083

Table S2. Bandgap parameters of the tested g-C_3N_4 samples

Sample	Valence band (VB)/eV	Conduction band (CB)/eV	Bandgap width/eV
B-CN	1.50	-1.14	2.64
G-CN	1.44	-1.23	2.67
0.2-P1-CN	1.44	-1.26	2.70
0.8-P1-CN	1.44	-1.27	2.71
3.2-P1-CN	1.44	-1.30	2.74
0.2-P2-CN	1.44	-1.18	2.62
0.8-P2-CN	1.44	-0.98	2.42
3.2-P2-CN	1.44	-0.69	2.13

Table S3. Summarized gas chromatography original data for the H_2 evolution experiments

Catalyst	B-CN		G-CN		1.6-P1-CN		0.8-P2-CN	
Time/Adjusted amount	H_2	Argon	H_2	Argon	H_2	Argon	H_2	Argon
1 h	2.8294	11.0331	4.4801	9.7736	6.9776	11.4489	9.359	7.2056
2 h	4.4646	10.5784	8.8434	9.4549	12.6437	9.6589	17.1389	7.0418
3 h	6.0686	10.4729	13.4498	9.4711	18.9701	10.4857	23.9577	6.6532
4 h	8.2131	10.5916	16.6306	8.7653	22.8219	8.8249	26.1052	5.4594
5 h	9.7681	10.6007	19.2148	7.9539	26.2269	8.4473	33.4608	5.8999

Table S4. H_2 evolution activity and AQY comparisons with the previously reported work

Catalysts	Usage/cocatalyst loading	Solution	Light source	H_2 evolution rate	AQY	References
g-C_3N_4 (Urea)	20mg/3.0 wt.% Pt	TEOA	≥395nm	~400 μmol·h^{-1}	26.5%@400nm	*Angew. Chem. Int. Edt.* **2014**, *53*, 9240-9245
g-C_3N_4 nanosheets	50mg/3.0 wt.% Pt	10 vol.% TEOA<br>10 vol.%+K_2HPO_4	≥420nm	~310 μmol·h^{-1}<br>947 μmol·h^{-1}	45.7%@380 nm<br>26.1%@420nm	*Angew. Chem. Int. Edt.* **2015**, *54*,13561-13565
N-doped g-C_3N_4	50mg/3.0 wt.% Pt	10% vol. TEOA	≥420nm	~78 μmol·h^{-1}	------	*Carbon*. **2016**, *99*, 111-117
Carbon dots/g-C_3N_4 nanosheets	50mg/0.2 wt.% Pt	5 vol.% Methanol	≥420nm	88.1 μmol·h^{-1}	------	*Appl. Catal. B: Environ.* **2016**, *193*, 248-258.
CoP/g-C_3N_4	50mg/3.0 wt.% CoP	15 vol.% TEOA	≥420nm	~97μmol·h^{-1}	12.4%@420 nm	*Adv. Funct. Mater.* **2017**, *27*, 1604328
P doped and tubular g-C_3N_4	100mg/1.0 wt.% Pt	25 vol.% Methanol	≥420nm	67 μmol·h^{-1}	5.68%@420nm	*Angew. Chem. Int. Edt.* **2016**, *55*, 1830-1834
P-TCN	100mg/1.0 wt.% Pt	20 vol.% Methanol	≥420nm	~59 μmol·h^{-1}	------	*Appl. Catal. B: Environ.* **2017**, *218*, 664-671.
PTCN-3 (P-dope g-C_3N_4)	50mg/3.0 wt.% Pt	10 vol.% TEOA	≥420nm	~10 0μmol·h^{-1}	4.32@420 nm<br>3.58@450 nm<br>1.28@500 nm	*Appl. Catal. B: Environ.* **2019**, *241*, 159-166.
P doped g-C_3N_4	50mg /3.0 wt.% Pt	10vol.% TEOA	≥400nm	195.8μmol·h^{-1}	6.1%@420 nm	*Small*. **2016**, *32*, 4431-4439.
S, P-doped g-C3N4	10mg /3.0 wt.% Pt	10vol.% TEOA	≥400nm	~50μmol·h^{-1}	18.93%@420 nm	*Int. J. Hydrogen Energ.* **2019**, *44, 20042-20055*
P, Na-doped g-C_3N_4	50mg/1.0 wt.% Pt	25 vol.% Methanol	300 W Xe lamp	191 μmol·h^{-1}	------	*J. Power. Source.* **2017**, *351*, 151-159
Protonated g-C_3N_4	100 mg/3.0 wt.% Pt	10% vol. TEOA	≥420nm	~28 μmol·h^{-1}	------	*Appl. Surf. Sci.* **2014**, *295*, 253-259
N deficient g-C_3N_4	10mg/1.0 wt.% Pt	25 vol.% lactic acid	≥420nm	69 μmol·h^{-1}	------	*Adv. Mater.* **2017**, *29*, 1605148
0.8-P2-CN (N deficient and protonated g-C_3N_4 nanosheets)	15 mg/1.5 wt.% Pt	20 vol.% TEOA<br>20 vol.% TEOA+K_2HPO_4	≥400nm	255.3 μmol·h-1<br>881.7 μmol·h-1	10.7%@420 nm<br>40.4%@420 nm	**This work**

Table S5. AQY measurement results of 0.8-P2-CN under different wavelengths

Wavelength (nm)	H_2 evolution rate (μmol/h)		Light intensity (mW/cm^2)	AQY (%)	
	TEOA	TEOA+K_2HPO_4		TEOA	TEOA+K_2HPO_4
400	102.7	319.3	3.45	14.9	46.3
450	72.7	209.2	3.24	9.9	28.6
500	58.7	133.4	2.97	7.9	17.9
550	12.4	112.9	2.65	1.7	15.5
600	23.3	125.3	2.79	2.7	14.9

15. Supporting Videos

Video S1. Stable and remarkable H_2 evolution over the 1.5 wt.% Pt-loaded 0.8-P2-CN nanosheets in the optimum TEOA/K_2HPO_4 mixture solution under visible-light irradiation (λ≧400 nm).

Video S2. Observable H_2 evolution over the spent 0.8-P2-CN photocatalyst (collected and washed after 25 hours irradiation, then dried at 40°C, 5 mg) in the optimized TEOA/K_2HPO_4 mixture solution.

Video S1 and **S2** can also be found with the following URL:

Video S1: https://www.dropbox.com/s/or6r0otrtmvadh8/Video%20S1.avi?dl=0
Video S2: https://www.dropbox.com/s/xpu7gzp3l087gvo/Video%20S2.avi?dl=0

References

[1] J. J. Zhang, W. S. Li, Y. Li, L. Zhong, C. J. Xu, Appl. Cata. B- Environ. 217 (2017), 30-36.
[2] P. Niu, L. C. Yin, Y. Q. Yang, G. Liu, H. M. Cheng, Adv. Mater. 26 (2014), 8046-8052.
[3] H. S. Tao, U. Diebold, N. D. Shinn, T. E. Madey, Surf. Sci. 312 (1994), 323-344.
[4] G. Kresse, D. Joubert, Phys. Rev. B 59(1999), 1758-1775.
[5] G. Kresse, J. Furthmüller, Phys. Rev. B 56(1996), 11169-11186.
[6] B.V. Lotsch, M. Doblinger, J. Sehnert, L. Seyfarth, J. Senker, O. Oeckler, W. Schnick, Chem. Eur. J. 13(2007), 4969-4980.
